# Doenças de africanos, doença africana: transformações da quijila, entre a África centro-ocidental e as Minas Gerais, Brasil, séculos XVII e XVIII

**DOI:** 10.1590/S0104-59702023000100052

**Published:** 2023-10-23

**Authors:** Junia Ferreira Furtado

**Affiliations:** i Programa de Pós-graduação em História Universidade Federal de Minas Gerais Brasil Professora, Programa de Pós-graduação em História/Universidade Federal de Minas Gerais e Universidade Federal de Ouro Preto.Belo Horizonte – MG – Brasil juniaf@ufmg.br; Universidade Federal de Ouro Preto Belo Horizonte MG Brasil juniaf@ufmg.br

**Keywords:** Quijila, Doença, África, Minas Gerais, Escravidão

## Abstract

Este artigo discute a origem da quijila/kijila na cultura centro-ocidental africana, mais particularmente no universo cultural dos imbangalas (jagas) e das populações ambundos e kimbundos, que viviam nas regiões portuguesas de Angola e do Congo, nos séculos XVII e XVIII. Em seguida, investiga como foi estruturado, compreendido e transformado o conceito de quijila tanto na África, basicamente um interdito alimentar, mas cujos significados e aplicações variam, quanto no Brasil, para onde foi transportado nos Setecentos, transformando-se numa doença que atacava os negros, especialmente os africanos de diversas origens, sendo enquadrada pelos médicos locais no universo da medicina hipocrática-galena vigente na época.

Em 1749, foi publicado, em Lisboa, um opúsculo, sem autoria, intitulado *Prodigiosa lagoa descoberta nas Congonhas das Minas do Sabará* ([Cialli], 1749; Furtado, 2022a, p.113-148). O tema deste artigo é uma instigante doença aqui descrita, chamada de quijila/kijila, e seu objetivo é buscar sua origem na cultura centro-ocidental africana, mais particularmente no universo cultural banto, vivenciado pelas populações jagas, ambundo e kimbundu, nas regiões portuguesas de Angola e do Congo. Nos séculos XVII e XVIII, a quijila se transformou na África, até que, no Brasil setecentista, mais especificamente em Minas Gerais, passou a ser uma doença que atacava somente os negros, especialmente os africanos de diversas origens, sendo enquadrada no universo hipocrático-galeno característico da medicina da época.

*Prodigiosa lagoa* relata a descoberta do poder curativo das águas da chamada lagoa Grande, na comarca de Sabará, em Minas Gerais, hoje lagoa Santa, e as curas de vários enfermos que, nos primeiros meses de 1749, beberam ou se banharam em suas águas. Finaliza com uma longa lista que relaciona os 109 primeiros doentes que tomaram os banhos e cujas enfermidades foram curadas ou mitigadas, indicando seus nomes, moradas “e qualidade de queixa” ([Cialli], 1749, p.12-27). A esses podem ser agregados mais quatro, citados no histórico, totalizando 113 doentes.

O texto atribui como elementos prodigiosos da água o aço e o vitríolo. O vitríolo, um sal mineral, semelhante ao vidro, atuava nas externas e era utilizado como antídoto e na cicatrização de chagas, inclusive dos membros viris. Sua presença na água curou “todas as queixas cutâneas”, bem como chagas abertas, “tumores, hérnias, verrugas” e dores. A quijila aparece incluída junto a sarnas, lepras, morfeias (lepra), formigueiros (feridas cutâneas, no formato dos buracos de formigas), erisipelas, e todos os tipos de chagas ([Cialli], 1749, p.9; Cialli [Romano], c.out. 1749, f.6-6v, f.7), considerados doenças externas.

## Escravos doentes, doenças de escravos

Entre os 113 enfermos, 88 eram homens (77,9% do total) e 25 eram mulheres (22,1%). No cômputo geral, 50 eram escravos(as) e 13 eram forros(as), equivalendo a 63 pessoas de cor, ou 55,75% dos doentes. Entre eles, estavam os seis doentes atacados pela quijila. Todas as doenças foram descritas e classificadas a partir dos conceitos vigentes na medicina hipocrático-galênica, sendo a quijila a única que atacou apenas negros (Nogueira, jan.-jun. 2011, p.47), as demais não distinguiram populações brancas das de cor. Mas, devido às péssimas condições de vida e de alimentação a que eram submetidos os cativos, as doenças eram mais recorrentes ou mais graves entre eles. Como exemplos, Pedro, escravo de Luiz Cardoso, no Caeté, depois de dois banhos expeliu “três lombrigas pretas de três palmos cada uma”, o que lhe causava “grandes dores na barriga”. Maria, escrava de Francisco Fernandes Braga, apresentava “um papo havia anos”, como se denominava o bócio. Acidentes de trabalho, especialmente na mineração, eram inevitáveis, e Pedro, escravo de Alexandre Teixeira, tinha uma chaga aberta, havia um ano, depois de ter caído sobre seu pé “um grande pau”; enquanto Ignácia, escrava de Brites Correia, apareceu com tosse e dor no peito, “lançando algum sangue”, depois de “lhe haver caído sobre os peitos, haveria quatro meses, uma gamela de roupas” ([Cialli], 1749, p.13, 16, 18-19).

Ao jogar luz sobre as doenças que acometiam os cativos, desnudando suas condições de vida, o texto de *Prodigiosa lagoa* é de grande interesse para a história da escravidão, das ciências e da medicina. No entanto, o estudo da quijila representa um desafio maior, pois não era apenas um mal recorrente entre os escravos, mas uma doença cujas origens podem ser traçadas a partir das cosmologias dos povos chamados de bantos,^1^ da África centro-ocidental, especialmente das populações jagas, ambundo e kimbundu.

Ainda que vários outros doentes apresentassem sintomas muito semelhantes aos que o médico italiano Antonio Cialli, autor do opúsculo (Furtado, 2022b, p.79-112), diagnosticou como quigila (com “g”), nenhum branco sofria do mal. É reveladora a comparação entre os casos de Antonio, escravo de Manoel Teixeira Lombo; de João de Araújo, filho do último; e de João da Costa Ferreira. A doença do primeiro atacara-lhe os pés e as mãos, que ficaram aleijados e estavam cobertos de chagas, o que lhe custara alguns dedos. João de Araújo, branco livre de 18 anos, também apresentava o corpo coberto de chagas e as pernas entrevadas devido aos estragos feitos pelas feridas que as cobriam. O mesmo acontecia com João da Costa Ferreira, branco, de 13 anos, filho de Manuel Jorge da Costa Ferreira, que sofria “com duas chagas em uma perna” e tinha “o dedo do polegar do pé direito comido de outra” ([Cialli], 1749, p.12). Apesar de o mal e os sintomas serem semelhantes, a quijila só foi diagnosticada no escravo, e não foi aventada para os dois brancos. Depreende-se que a presença de feridas que não cicatrizavam e de inflamações nos membros, levando à perda de dedos, não foi determinante no diagnóstico da quijila e, sim, a qualidade do doente, isto é, o fato de ser negro.

## A quijila e os jagas

Desde o século XVI, os profissionais da saúde luso-brasileiros buscavam incorporar à matéria médica os conhecimentos indígenas e africanos sobre as plantas nativas dos Novos Mundos, e os tratados médicos enquadravam as doenças, mesmo as que acometiam as populações autóctones e as escravizadas no Novo Mundo, no interior da nosologia própria da medicina hipocrático-galênica europeia (Furtado, 2007, p.127-151). Mas não foi o que ocorreu nas páginas de *Prodigiosa lagoa*. De forma inédita, uma doença – a quijila –, cujos significado e origem se dão no interior da cultura e das crenças africanas, é que foi incorporada ao quadro nosológico europeu. Se os sintomas não foram, por si só, capazes de levar ao seu diagnóstico, para compreender a doença é necessário buscar sua origem no interior da cultura centro-ocidental africana, compartilhada pelos que sofriam do mal.

Os três tratados que Antonio Cialli escreveu sobre as curas que observou com o uso das águas da Lagoa Grande configuram casos únicos e raros de textos de medicina erudita da época que fazem referência a uma doença de origem africana ([Cialli], 1749; Cialli, 16 jun. 1749; Cialli [Romano], c.out. 1749). Dessa forma, o estudo do mal e de suas origens contribui para elucidar aspectos próprios da cultura dos escravizados em Minas Gerais, na América portuguesa, que foram, em geral, apagados, devido ao caráter hegemônico da cultura europeia em terras brasílicas, especialmente no que diz respeito às doenças e suas práticas de cura.

Comecemos com a origem do termo. Em kimbundu, língua falada pelas populações ambundo de Angola, o sentido básico de kijila é proibição ou ainda tabu. A palavra aparece muito cedo nos catecismos traduzidos para o kimbundu, como no de Francesco Pacconio e António Couto, publicado em Lisboa, em 1642 (Pacconio, Couto, 1642), sendo usada para traduzir os tabus impostos pelos “ídolos”. Na edição de 1645, é também empregada para se referir a um dos Dez Mandamentos, revelados por Deus a Moisés, substituindo o termo “milongo”, mais secular, que passou a ser empregado somente para se referir às leis ou aos decretos emitidos pelo rei. Fica clara a distinção semântica do sentido dos dois termos: kijila circunscrevendo-se às regras impostas no universo propriamente religioso, e milongo, no secular.

A palavra quijila ou kijila aparece diretamente ligada aos costumes do grupo que os portugueses chamaram de jagas mas, originalmente, não se referia a um mal ou doença específica. Era cada uma das 14 leis ou proibições e rituais seguidos por esse grupo, que habitava o interior distante de Luanda, que a etno-história moderna identifica como imbangalas, sobre quem recaía a acusação de ser sangrentos selvagens antropófagos, que viviam da razia das populações ambundo do entorno (Miller, 1973, p.121-149; Souza, 2013, p.141, 2018, p.98). A identificação e a origem dos jagas pela historiografia sobre a África, a partir das crônicas europeias do século XVII, continuam controversas. O temo foi usado para identificar guerreiros nômades e selvagens que, nessa época, faziam incursões violentas ao Congo e arredores e inspiravam o terror a europeus e africanos, não constituindo, incialmente, uma identidade étnica em particular. Tudo indica que jaga derivava do vocábulo aka, que significava “o outro”, e que era empregado a vários grupos considerados pelos povos locais de origem ambundo, aliados dos portugueses, como salteadores, atacantes ou estrangeiros e que tinham feito da mobilidade e da guerra seu modo de vida. Alguns cronistas europeus da época identificaram sua origem nos zimbas e mumbos, povos da costa leste, que teriam se deslocado para o oeste nessa época, o que se fez pela prática comum da antropofagia e do nomadismo. *Grosso modo*, os jagas passaram a ser descritos, nas fontes e na literatura europeias, como nômades e que jamais construíram um Estado coeso. Eram antropófagos, viviam da razia e da guerra contra os povos bantos locais e resistiam à penetração e à colonização dos portugueses. Opunham-se particularmente aos ambundo, mais numerosos, que eram sedentários, viviam da agricultura e do pastoreio e tinham sua organização sociopolítica assentada na linhagem, constituindo Estados, sendo o reino mais importante na região o Ndongo ou Dongo (Miller, 1973, p.121-149; Thorton, 1978, p.223-227; Hilton, 1981, p.191-202; Pinto, 1999-2000, p.193-243; Souza, 2018; Furtado, 2021, p.442-455).

Pelo fato de a coesão sociopolítica dos jagas não se assentar em linhagens e por nunca terem desenvolvido um Estado, a adesão e o pertencimento ao grupo eram marcados “por uma série de ritos e pela observação de regras específicas” (Souza, 2018, p.121), as ijila (plural de quijila), conjunto de regras ou interdições. Inicialmente, muitas das ijila “tinham a ver com o papel das mulheres” e “a maioria das outras tratava dos rituais realizados em funerais, promoções ou mudanças de posição social, e mecanismos para iniciar crianças pequenas no quilombo”. Entre os ambundos, o chamado kilombo era “uma sociedade de iniciação ou campo de circuncisão, onde os jovens do sexo masculino eram preparados para o *status* de adulto” (Schwartz, 2001, p.249-255). Os jagas adotaram esse tipo de organização, baseando sua identidade na valentia individual, e não na linhagem, pois reuniam indivíduos de diferentes origens. No século XVIII, o termo kilombo passou a ser empregado pelos portugueses para identificar as capitais dos reinos nativos, especialmente dos jagas, que resistiam ao seu avanço no centro-oeste africano e, no Brasil, em fins do século XVII, generalizando no XVIII, as comunidades de escravos fugidos (Lara, s.d., 2021).

Um dos grandes informantes e divulgadores dos costumes e da barbárie dos jagas foi o padre capuchinho italiano Giovanni Antonio Cavazzi (de Montecuccolo), que, em 1687, depois de missionar em Angola, publicou a *Istorica descrittione de tre regni Congo, Matamba et Angola*. Segundo ele, a quijila (empregando o nome no singular) era um conjunto de leis que foi instituído inicialmente pela rainha imbangala Temba-Ndumba, “aplaudida e respeitada como mulher de extraordinária coragem”, e “dividem-se em domésticas, religiosas e civis” (citado em Leguzzano, 1965, v.2, p.177-179). As domésticas prescreviam “a observância de algumas tradições dos antepassados, como a abstinência da carne de porco, de elefante, de serpente e de outros animais”, embora assegure que “isso tudo implique uma grande violência ao natural apetite que todos têm”, devido à antropofagia que praticavam (citado em Leguzzano, 1965, v.1, p.179-181).

De forma preconceituosa, a partir do ponto de vista católico, Cavazzi considerou-as “ridículas e supersticiosas”, afirmando que essas leis “têm por objeto algumas prescrições que, de um momento para o outro, são inventadas pelos feiticeiros astuciosos e perspicazes, conforme a oportunidade e o gênio daqueles desgraçados” (citado em Leguzzano, 1965, v.2, p.180). Feiticeiros é como se refere, pejorativamente, aos ngangas ou gangas, como eram chamados os sacerdotes africanos que, segundo ele, “tratam da vida privada, das contendas, das doenças, dos perigos de morte e de outros assuntos semelhantes” (citado em Leguzzano, 1965, v.1, p.180). Para ele, a quijila era a negação dos princípios cristãos e símbolo da barbárie dos jagas. Também repercutia a visão que os próprios ambundos tinham desses mandamentos, que consideravam inumanos, já que na cosmologia Mbundu eles eram considerados cruéis.

A partir da descrição do capuchinho, percebe-se que a interdição alimentar não correspondia ao conjunto das ijila, mas era uma das quijila domésticas, imposta pelos sacerdotes e transmitida pelos antepassados por meio da tradição oral. Interditada era “a carne de porco, de elefante, de serpente e de outros animais” (citado em Leguzzano, 1965, v.2, p.179), mas Cavazzi não especifica quais penalidades sofriam os infratores nem que a desobediência provocasse alguma doença. A proibição de ingestão de alguns animais, especialmente os de grande porte, não foi exclusiva dos povos africanos, mas recorrente em várias culturas, inclusive na europeia do século XVIII, nas quais adquiria funções diversas, como a reserva de carne de alguns animais, principalmente os de grande porte, a alguns indivíduos, geralmente de posição hierárquica superior, como os reis (Thompson, 1987).

As ijila foram adotadas por parte das populações ambundo a partir do reinado da famosa rainha Jinga ou Njinga, que, em 1624, reivindicou para si o trono do Ndongo. Lutando contra os portugueses, que tinham o apoio de alguns grupos aliados de jagas (que imediatamente abandonavam as ijila), a rainha estabeleceu uma aliança, em 1629, com o chefe imbangala Cassa, com quem acabou por se casar, adotando as tradições jagas e assumindo a chefia do grupo. Acuada pela guerra, em 1630, acabou por estabelecer seu reino em Matamba, a oeste do rio Cuanza, de onde comandou a resistência aos portugueses até 1656, quando estabeleceu um tratado e se reconverteu ao catolicismo, que professara na juventude.

Sem nunca abandonar a linhagem como identidade do grupo, inclusive levava consigo uma urna funerária com os ossos de seus antepassados, Njinga também adotou os preceitos e as interdições da ijila (Heywood, 2019, p.125-128, 197-227; Souza, 2018, p.110-114, 128-130). Entre todos os líderes locais, ela ocupou papel especial pela resistência que manteve no interior, entre o Ndongo e Matamba, “passando a viver conforme as leis kijila e junt[ando] em torno de si os que resistiam à conquista portuguesa”, fossem jagas ou ambundos e, de fato, “ser jaga não eliminava uma possível origem étnica ambunda” (Souza, 2018, p.128, 152). O que se observa é que, para os colonizadores, os jaga e a ijila se tornaram associados à antropofagia, à selvageria e à barbárie, e eram atributos conferidos à rainha e a seus súditos. Foi a identidade com os primeiros e a adoção das últimas que permitiu que Njinga atraísse todos os que resistiam aos portugueses, independente da origem étnica ou da linhagem a que pertenciam, o que, na década de 1640, transformou Matamba no maior Estado africano da região e contribuiu para a maior difusão dos ritos das ijila entre a população local.

## Quijila e sua crioulização na África centro-ocidental

Em 1656, acuada pela guerra, a que resistia havia cerca de quarenta anos, Njinga estabeleceu a paz com os portugueses, devendo, entre outras imposições, abandonar as ijila, adotar o catolicismo e promover a conversão de seus súditos. Missionários capuchinhos italianos foram enviados para a ação missionária e buscaram erradicar elementos da cultura africana considerados heréticos e bárbaros. Entre eles, destaca-se Cavazzi (citado em Leguzzano, 1965, v.1, p.113), que advertiu em seus escritos que “esses pretos praticam diversas superstições”, que não “gostaria de narrar, pois são ridículas”, desculpando-se de que, se o fazia, era “apenas para instruir os missionários, para que possam tirar os Pretos dos seus enganos”. Entre esses enganos, sua maior condenação foi dirigida à quijila que atribuiu aos jagas.

Mesmo depois da chegada dos capuchinhos, Njinga se comportou de maneira paradoxal. Por um lado, transformou “Matamba [n]um centro de irradiação do catolicismo” (Souza, 2018, p.143), adotou o nome cristão de Ana de Sousa e buscou restringir a ação dos ngangas e dos xinguilas – os últimos estabeleciam as conexões com o mundo dos mortos –, tentando forçar seus súditos a abandonar os costumes e os ritos africanos. Por outro lado, sob pressão das elites e dos sacerdotes, costumes e ritos ambundos e imbangalas, inclusive as ijila, foram preservados, sendo importantes fontes de legitimação de seu poder. É o que se observa em seu funeral, assistido por Cavazzi, que teve que tolerar a alternância de ritos católicos, ambundos e jagas. Um tambo, cerimônia de enterramento jaga, foi realizado, tendo sido interditado pelo religioso apenas a prática de sacrifícios humanos (citado em Leguzzano, 1965, v.2, p.156-157, 161; Souza, 2018, p.143, 160, 167, 170).

Sob pressão dos religiosos, no seu reinado, “as leis kijila [teriam deixado] de vigorar, em favor das normas ambundas”, e “seu povo construiu novas identidades, resultantes das situações então vividas”, que combinavam “elementos tradicionais com as inovações introduzidas pelos missionários” (Souza, 2018, p.207-210). Com a morte da famosa rainha, a política de seus sucessores oscilou, tendendo a reprimir a prática do catolicismo e a resgatar os costumes africanos. Nas cerimônias do enterro da irmã de Njinga, batizada de d. Bárbara, que a sucedeu, por exemplo, o tambo voltou a ser celebrado com sacrifícios humanos (Leguzzano, 1965, v.2, p.168), e, no de Jinga-Mona, que reinou a seguir, os missionários acabaram sendo expulsos. Mas não se tratou apenas de reconstruir uma identidade ambunda, assentada na linhagem. Como no tambo de d. Bárbara, vários costumes jagas permaneceram, ainda que transformados. Vejamos o que se passou com a quijila nesse contexto de mudanças.

Em 1682, o padre capuchinho Girolamo Merolla da Sorrento chegou para missionar na região, publicando, dez anos depois, a *Breve e Succinta Relatione del Viaggio nel Regno di Congo*. Numa passagem que se refere ao Congo, ele menciona a quijila, que denomina cheguilla, como sendo o sexto juramento. No entanto, os juramentos que faziam os ambundos tinham natureza e funções distintas das ijila, sendo usados para arbitrar a justiça e se constituíam de várias provas a que o indivíduo era submetido para se comprovar a sua inocência ou não (Leguzzano, 1965, v.1, p.102-113; Ferreira, 2013, p.196-201). Aqui, é importante destacar que Merolla da Sorrento emprega uma forma cognata do termo, em kikongo, falado pelas populações da região do Congo/Kongo português, mas atribui ao termo o significado que quijila possuía em kimbundu, mesmo sendo provável que conhecesse as distinções com que a palavra era empregada nas duas sociedades. Segundo ele, pelo juramento, “como de costume dos pais ou dos feiticeiros d[ão] alguma regra aos filhos a ser obedecida inviolavelmente”, que consistia em “se abster de comer qualquer tipo de galinha, ou carne selvagem, ou frutas dessa espécie, ou raízes cruas, ou cozidas dessa maneira, ou de outra maneira, com diferença bestial” (Sorrento, 1692, p.146-147). Ou seja, trata-se apenas de uma interdição alimentar.

O padre, a quem tudo pareceu ridículo, espantou-se “que eles preferem jejuar por alguns dias do que provar as coisas proibidas”, pois “têm de certo que vão morrer em breve”. Importante destacar que, para ele, quem dita o preceito é a mãe e somente se ela “não lhes deu Chegilla” é que “vão imediatamente para recebê-lo dos Magos (Maghi)”, pois “eles têm de certo que vão morrer em breve” (Sorrento, 1692, p.146-147). A referência aos ngangas como magos ou feiticeiros refletia a disputa entre eles e os missionários católicos pelo poder de comunicação com o sobrenatural. Em seguida, o religioso narra um caso exemplar de “um preto” que, em viagem, foi servido de uma galinha do mato por seu anfitrião que, apesar de perguntado, lhe sonegou a informação sobre a origem selvagem do animal. Quatro anos depois, ao encontrar novamente esse anfitrião e ser informado da verdadeira origem da galinha, o convidado concluiu que “tinha Chegilla” e “ficou tão triste que não conseguiu sobreviver mais de vinte e quatro horas”, pois “tais pessoas, às quais têm por certo que se transgredirem a Chegilla ocorre súbito a morte” (p.146-147).

A descrição que Merolla da Sorrento faz da quijila se diferencia da informada por Cavazzi em alguns aspectos: (1) não se refere mais a uma das regras das ijila, mas de um juramento; (2) se limita apenas a uma interdição alimentar; (3) a transmissão principal da interdição é feita pela mãe e, somente na ausência dela, pelos sacerdotes nativos; (4) os alimentos interditados diferenciam-se um pouco, e foram acrescentadas prescrições referentes ao modo de preparo dos alimentos; (5) o desrespeito à interdição resultava na morte do transgressor. Como se sabe que esse religioso foi leitor atento do livro de Cavazzi, que serviu de inspiração para o seu, essas diferenças não podem ser creditadas a seu desconhecimento da obra ou do kikongo ou ainda à dificuldade de compreensão do que observou, mas às transformações que se operavam na cultura local sob o impacto do catolicismo e, principalmente, sob a progressiva hegemonia dos costumes ambundo sobre os jagas entre os bantos angolanos e mesmo entre os congoleses, pois trocas culturais entre esses povos eram frequentes. Os missionários foram implacáveis com os sacrifícios humanos, que constituíam parte importante das ijila, e perseguiam e deslegitimavam o poder dos líderes religiosos africanos.

Vários historiadores da África utilizam o conceito de crioulização (Mintz, Price, 1976, 2003) como ferramenta teórica para compreender as transformações operadas na cultura africana centro-ocidental sob o impacto do cristianismo e das instituições sociopolíticas introduzidas pelos portugueses, ainda que nem sempre concordem com seu significado. Para James Sweet (2007), esse conceito não faria sentido para sociedades do interior do continente, não impactadas pelo tráfico atlântico. Já para Linda Heywood (2008), entre outros, seu emprego é válido tanto na costa quanto no interior, sendo que a suas análises também se centram na miscigenação entre a cultura portuguesa e a africana (Ferreira, 2013; Schleumer, 2018; Lara, 2021, p.140-152), pressupostos teóricos que podem ser instrumentalizadas para o estudo das transformações pelas quais a quijila passava no complexo Angola-Congo.

No entanto, as mudanças operadas ainda na África centro-ocidental e em curto espaço de tempo somente em parte podem ser creditadas à ação dos missionários católicos, que podiam ser condescendentes com as restrições alimentares, mas não com os sacrifícios humanos das ijila dos jagas. O fato de o principal elo de transmissão ter se transferido, conforme atesta Merolla da Sorrento, dos ngangas para a mãe, não ocorreu somente pelo impacto da ação dos religiosos cristãos, pois é também um reflexo da progressiva hegemonia da cosmologia ambundo sobre as comunidades jaga-ambundos, que reuniam os que resistiam, no interior de Angola, aos portugueses. Para os ambundos, “o culto da ancestralidade tinha papel específico de articular as relações sociais no interior do grupo de parentesco (as linhagens)” (Marcussi, 2015, p.153), e eles também viam com reservas os aspectos inumanos dos sacrifícios jagas. Por sua vez, a presença do conceito de quijila entre as populações kimbundu, do Congo, pode ser explicada pelas trocas culturais que se davam entre as populações centro-ocidentais africanas, o que se intensificou após a chegada dos portugueses. O termo crioulização pode ser articulado para analisar essas transformações, mas com um sentido mais amplo. Ajuda a elucidar tanto o impacto dos lusitanos, do cristianismo e do tráfico de escravos quanto a incorporação de práticas culturais de diferentes grupos locais, caso dos jagas, ambundos e kimbundus, que se amalgamavam e se transformavam, respondendo aos anseios do dia a dia impostos sobre suas sociedades nativas, especialmente pelos europeus.

## A quijila e a cura do mal do quibuco

O manuscrito português anônimo “Ritos gentílicos e superstições que observam os negros do gentio desse reino de Angola desde o seu nascimento até a morte”, que não está datado, fornece outras pistas do processo de crioulização que, ainda na África centro-ocidental, se operava no conceito de quijila. Entre outros tópicos, descreve as enfermidades e os ritos de cura vigentes em Angola que, como se observa pelo título, são descritos a partir do ponto de vista do seu autor anônimo, que os qualifica sempre de forma negativa, pejorativamente classificando-os como superstições e ritos gentílicos, isto é, pagãos ou idólatras.

Chama a atenção no texto o fato de que o papel dos ngangas seja apontado como restrito à cura das doenças, sendo eles descritos como um misto de feiticeiros e cirurgiões. É o último termo que o autor encontra, na cultura europeia, para, por analogia, identificá-los. Fica evidente ao leitor europeu que, como cirurgiões, eles eram detentores de um saber prático, sendo pouco instruídos e mal preparados, o que implicava a sua desqualificação em relação aos médicos, únicos detentores do saber erudito. O uso do termo cirurgião e a forma como seu papel é descrito obliteram o importante papel religioso e na transmissão dos costumes e dos ritos dos antepassados que os ngangas ainda desempenhavam na África. No texto, sua ação foi circunscrita a cuidar dos doentes e a prescrever os tratamentos costumeiros, pois, sob a pressão dos portugueses, nas questões espirituais, eram cada vez mais substituídos pelos padres católicos ou tinham que camuflar sua ação espiritual sob as práticas curativas.

Segundo os *Ritos gentílicos...* (s.d., f.2), “quando algum [indivíduo] padece enfermidade com lesão no seu entendimento se diz ter quilundos, [e] para estes se curarem consultam a um cirurgião chamado nganga de quilundos, ... e o dito cirurgião se recolhe para outro quarto sem pessoa alguma, aonde invoca ao diabo, com quem consulta a enfermidade”. Uma vez “havida a saúde, se faz festa ao quilunfo, que é o ídolo invocado com muita comiraina [*sic*, comida] em ação de graças”. Para o autor anônimo, medicina popular e feitiçaria se consubstanciavam na prática dos ngangas, que invocavam, para realizar a cura, não só seus ídolos, mas o próprio diabo em pessoa, o que significa a leitura de seus ritos e de suas crenças a partir de um ponto vista católico, condenando-as, já que idolatria e demonologia marcariam suas práticas de cura. Os africanos são vistos como o outro, e sua religião, como coisa do diabo (Schleumer, 2018).

Entre as enfermidades que o texto descreve, “há outra doença que chamam de mal de quibuco, para cuja cura se consulta ao ídolo quibuco”, sem explicar quais os sintomas e que parte do corpo afetava. No manuscrito, quibuco é tanto o ídolo que se consulta quanto a enfermidade para a qual se busca a cura, que era um dos “achaque[s] de imaginação” que os negros padecem (Ritos gentílicos..., s.d., f.2). O tratamento consistia no sacrifício de cabritos e galinhas, oferecidos ao ídolo que os ngangas invocavam. Uma vez recuperada a saúde, “o cirurgião ... dava aos tais curados seus preceitos de comer ou não comer isto ou aquilo, os quais preceitos se chamam Quigilles, que observam a risca” (f.2). Quijila continua a se referir aos “preceitos de comer ou não comer isto ou aquilo”, mas que, agora, são restritos ao período de convalescença de uma doença específica, o quibuco, e prescritas pelo nganga, recuperando, em parte, seu papel de intermediário com o sobrenatural, mas restrito ao universo de cura das doenças.

Ainda que o texto não esteja datado, é possível perceber que novas transformações se operavam. Desconectada das ijila, das quais preservava apenas o nome, a quijila não era também uma regra nem uma interdição alimentar cotidiana ampla, passando a se referir à dieta alimentar, prescrita pelos ngangas, apenas depois da cura do mal do quibuco. Pela primeira vez, é associada a uma doença específica, mesmo não sendo o mal em si, mas não são descritas as consequências da quebra da dieta. Dessa maneira, não mais se tratava exatamente de um tabu.

Acima de tudo, essa descrição da quijila revela que, com o tempo, se tornara um rito observado por todos “os negros do gentio desse reino de Angola”, não sendo restrita aos jagas, nem mesmo circunscrita ao reino de Matamba. Despojada do sentido original, disseminara-se pelas populações ambundo da região da Angola portuguesa. Tal expansão revela, mais uma vez, a crioulização pela qual passavam as diferentes cosmologias dos povos bantos locais, que intercambiavam entre si ritos e crenças. Uma chave para entender essa consubstanciação e sua disseminação é a afirmação de Cavazzi de que, depois da morte de Nzinga e de d. Bárbara, o sucessor Jinga-Mona invocou em praça pública, por meio dos xinguilas, o espírito da primeira, reencenando a importância da linhagem para estruturar seu poder. Mas, em seguida, “mandou que fossem sacrificados todos os prisioneiros da expedição anterior” e “que fosse aberto o ventre a muitos dos presentes e que, com seu sangue, fossem borrifados os soldados e ele mesmo, fazendo alegres votos de prosperidade” (citado em Leguzzano, 1965, v.2, p.170). Nesse caso, era uma reencenação da quijila mais importante dos jagas, a instituída pela rainha Temba-Ndumba, no que foi seguida por seus sucessores, inclusive Nijinga, que consistia em untar o corpo, antes da guerra, com um unguento mágico que tinha como principal ingrediente o corpo de crianças sacrificadas. Observa-se que Jinga-Mona legitimava seu poder na encenação de ritos importantes para os ambundos e jagas e, depois da cerimônia, mandou “mensageiros por todo o reino para que ... fossem renovadas as antigas cerimônias e que, portanto, cada um pudesse viver livremente conforme os ritos dos Jagas ... e sem impedimento nenhum reintroduziu os antigos ritos” (citado em Leguzzano, 1965, v.2, p.170).

## A quijila como maldição

No começo do século XVIII, segundo o dicionário de português, publicado em Lisboa em 1720, por Rafael Bluteau, a quigila (escrita no verbete com “g”) passou a ser uma “maldição, que os pais dos negros de Angola dão aos filhos, dizendo-lhes que se comerem veado, carneiro etc. lhes dão a sua maldição, e dizem que, comendo, lhes veem umas nódoas, ou outros sinais, e morrem” (Bluteau, 1720, p.58). Mudanças aconteceram mais uma vez no seu significado ou pelo menos no entendimento que os europeus, mais particularmente os portugueses, tinham da quijila africana. A interdição alimentar, presente no tabu original, continuou a ser mencionada, porém o termo se refere não propriamente a ela, mas à maldição causada pela quebra da dieta, ainda que Bluteau não se refira a essa maldição como uma doença específica.

O termo maldição circunscreve a compreensão da cultura africana como feitiçaria, tal qual era próprio dos europeus, que ecoavam os missionários católicos, intolerantes com seus ritos e crenças. Havia por parte da igreja um modelo no qual “senhores e escravos, brancos e negros deviam ser antes de tudo cristãos ... Nesse modelo, não havia tolerância com as práticas de origem africana, vistas como demonizadoras” (Souza, 2002, p.229). A visão sobre o continente é marcada pelo negativo, pela intolerância em relação aos hábitos, às religiões e aos seus ritos, aos costumes da terra, entre outros, e alteridade é o que passa a marcar a relação dos europeus com a África.

Bluteau (1716, p.734) descreve como consequência da desobediência o aparecimento das “nódoas, outros sinais e morte”. Nódoas, segundo o mesmo dicionário, eram pintas que surgem principalmente no rosto, e o que se observa é que, apesar do destino fatal que aguarda o transgressor, as nódoas são menos sintomas de uma doença, e mais marcas e sinais públicos, impostos pelos pais, que tornavam evidentes para a comunidade a transgressão praticada pelos filhos. Tal entendimento explica por que, para Cialli, “as nódoas negras”, que apareceram “no rosto, braço e pernas” de Francisco Moura Chagas, nos quais “em que a pouco e pouco ia perdendo também a sensação”, não foram diagnosticadas como quijila. Esse doente era branco e livre, perfil inverso dos acometidos de quijila, e o médico creditou seu mal ao fato de, “há três anos, havendo-se molhado, lhe sobrevieram umas dores na mão e perna direita, da qual perdeu logo pela parte de fora a sensação” ([Cialli], 1749, p.23).

Para Bluteau, no início do século XVIII, a crença na quijila era compartilhada por todos os africanos de Angola, sem distinguir o grupo a que pertencia, e incorporava elementos da cultura dos jagas, caso das restrições alimentares, e dos ambundo, como a importância conferida à linhagem – a maldição passara a ser imposta pelos pais, e não apenas pela mãe, como aparecera entre os kimbundos. Nesse sentido, ele retoma a informação de Merolla da Sorrento de que não era mais necessária a intermediação dos sacerdotes nativos, revelando o declínio do seu poder frente ao avanço do catolicismo na África, mas também nas Américas, onde os escravizados enfrentavam inúmeras dificuldades para transplantar intactos seus cultos, uma delas a ausência dos seus líderes religiosos. Também não há menção a ser uma dieta guardada durante o período de convalescença, nem qualquer relação com um culto em particular, caso do quibuco, como havia sido descrito nos *Ritos gentílicos...* Não é explicado, ou não sabe o dicionarista, o significado e a razão de tais proibições nem porque elas se limitam a apenas dois animais – veados e carneiros. A escolha deles parece ser resultado das disputas, na Europa, entre reis e súditos, e na América, entre senhores e escravos, pelo alimento de grande porte caçado nas matas e da necessidade de preservação dos rebanhos dos primeiros da fome cotidiana dos cativos. Nesse sentido, a menção aos carneiros e veados e à transmissão da maldição pelos pais, e não somente pela mãe, adiciona um ingrediente à crioulização da quijila, o tráfico atlântico de escravos e sua importação maciça para a América portuguesa, levando consigo um repertório de crenças e costumes já em transformação na África centro-ocidental e que, atravessado o Atlântico, sofria novas pressões por mudanças.

## Entre Angola e Minas Gerais, breve intervalo

As guerras que os portugueses desferiram no interior de Angola, ao longo do século XVII, contra os jagas e, em especial, contra a resistência que a rainha Njinga liderou no Ndongo e em Matamba, renderam muitos escravos ao tráfico atlântico. A partir de 1649, o fluxo de escravos recrudesceu depois da derrota e da assinatura do acordo de paz com Njinga, quando foram reabertas as feiras do interior de Angola, onde os lusitanos se abasteciam de cativos. Nessa ocasião, a rainha mandou de presente ao governador, ao bispo e ao ouvidor geral de Luanda, “algumas cabeças de escravos da gente fugida”, que viviam no reino há muito tempo, além dos 2 mil que enviou como resgate de sua irmã que estava aprisionada na capital (Souza, 2018, p.139-141, 155, 161). Devido à ação missionária dos capuchinhos, os ngangas apanhados praticando os antigos ritos jagas que haviam sido proibidos também eram destinados ao tráfico atlântico. Segundo Cavazzi (citado em Leguzzano, 1965, v.2, p.145-146), um deles “foi enviado com seus dois companheiros para as minas do Rio de Janeiro”; outro, “com toda a sua família”, foi condenado “às minas da América” e um terceiro “foi açoitado pelas ruas da cidade [de Santa Maria de Matamba] e, por fim, despachado, com outros companheiros para a América”. Referia-se à capitania de Minas Gerais, onde depósitos auríferos haviam sido encontrados.

A guerra aos que resistiam ao avanço lusitano estendeu a “fronteira de escravização” para o interior de Angola (Miller, 1988), e muitos dos recém-chegados à América eram destinados às Minas Gerais, onde a fome pela mão de obra escrava crescia na mesma proporção que aumentava, na capitania, a exploração de ouro e de diamantes. Calcula-se que, no início do século XVIII, cerca de 40% dos recém-chegados ao Brasil foram redirecionados para a área mineradora (Goulart, 1975, p.165), o que teria representado uma média de cerca de 1.560 cativos por ano (Ribeiro, 2005, p.195).

A colonização das Minas Gerais ocorreu num momento de mudança da direção do tráfico negreiro, até então dominado pela “onda angolana” (Miller, 1988; Lara, 2021, p.185-199). A partir do final do século XVII, a região da Costa da Mina começou a suprir, em número crescente, a demanda. Majoritariamente de origem sudanesa, na capitania, eram chamados de “minas”. Só no biênio “1725 e 1727, cerca de 5.700 cativos entra[ra]m, anualmente, no porto da cidade do Rio de Janeiro, procedentes da Costa da Mina e de Cabo Verde. Desses, 2.300 [foram] transferidos em seguida para as Minas” (Soares, 2000, p.77). No entanto, ao longo do século, os minas diminuíram, quando os bantos, oriundos do complexo Congo-Angola-Benguela, voltaram a chegar em maior quantidade. Cálculos realizados para o intervalo de 1720 a 1888, a partir de inventários *post-mortem*, apontam que 28,3% dos africanos eram de origem benguela, 23,9% de angola, 10,7% do congo e os minas representaram apenas 10,5% (Bergad, 1999, p.151).

A partir desse perfil da composição do tráfico e da constatação de que, por meio dos escravizados oriundos de Angola (e talvez também do Congo), suas crenças cruzavam o Atlântico, cabe inquirir como o conhecimento da quijila foi transmitido entre os cativos em Minas Gerais, que significado adquiriu entre eles e se foi compartilhado com escravos de outras origens. Os estudos sobre a escravidão no Brasil têm incorporado, de forma crescente, a problematização “da ‘sobrevivência’ das culturas africanas nas Américas e das ‘adaptações’ que elas haviam sofrido no novo contexto” (Lara, 2021, p.124). A exploração da área mineradora exigiu um contingente numeroso de escravos, uma comunidade heterogênea que, no ambiente da senzala, promoveu interações e reapropriações entre culturas distintas trazidas de suas terras natais, mas que, no contexto do cativeiro, se amalgamavam e eram moldadas por ele. Como adverte a historiografia recente, “a cultura africana era um elemento importante para se conhecer a lógica das ações dos escravos, mas havia de levar em conta a experiência da própria escravidão” (Lara, 2021, p.125). No cativeiro, onde africanos das mais diversas origens conviviam e intercambiavam crenças e ritos entre si, ocorreu uma “reconstrução, reinvenção ou reinstitucionalização das religiões africanas no Brasil” (Parés, 2007, p.109). Isso foi possível porque “os princípios cosmológicos fundamentais (explicação, previsão e controlo) partilhados pela maioria destes povos africanos permitiram-lhes elaborar concepções comuns” (Sweet, 2007, p.142), mesmo oriundos de diferentes regiões do continente.

Ao se aceitar a ocorrência de um processo de africanização nas senzalas mineiras, torna-se menos importante saber a origem dos negros acometidos de quijila que se banharam na lagoa. Cinco eram escravos e um era forro, sendo que só para dois deles há a certeza de serem africanos: o preto forro Francisco Xavier Barreto (caso 12), pois a designação de cor, preto(a), era atribuída, na capitania, aos importados da África via tráfico negreiro, diversamente dos crioulos, como eram chamados os nascidos no Brasil; e Manoel, escravo de Manoel Rodrigues (caso 75), pois contraíra a doença na África 17 anos antes de chegar ao Brasil ([Cialli], 1749, p.13, 21). No entanto, mesmo para os dois, não é possível saber de que parte do continente eram originários, se provinham do complexo Congo-Angola ou da Costa da Mina. Certo é que o termo negro era normalmente empregado na capitania para se referir aos escravos de origem africana, e o médico Antonio Cialli afirma tratar-se de uma doença que atacava somente os negros, isto é, o mais provável é que se referisse aos africanos. Se seu número parece reduzido (apenas seis doentes), a dicionarização do termo quijila e sua menção em outro documento produzido na capitania, analisado a seguir, revelam que se tratava de crença generalizada entre os escravos na capitania, ainda que seu significado se altere com o tempo.

Uma pista que a quijila fornece da “nova cultura, afro-americana, que nascia desse encontro de diversas heranças africana com a sociedade escravista do Novo Mundo” (Lara, 2021, p.125-126), aparece no *Compêndio narrativo do peregrino da América*, de Nuno Marques Pereira (1939), publicado em 1728. Nele, o autor, sob o ponto de vista católico, não se cansa de condenar os ritos e as cerimônias africanas que os escravos, não raro com a complacência dos senhores, praticavam na capitania. Acusa essa “gentilidade, que vem de Angola e [da] Costa da Mina, [de] haver entre eles aquele abuso das Quijilas” (com “j”), do que se infere que, em Minas Gerais, nesse momento, a crença passara a ser compartilhada por grupos bantos do centro-oeste e sudaneses da costa nordeste africana, capazes de estabelecer aproximações entre suas culturas de origem e encetar trocas e interações, a despeito das diferenças e particularidades existentes entre elas. Segundo o autor, ambos os grupos “guardam” a quijila, “alguns tão pontualmente, como se fora um Mandamento da Lei de Deus, e antes morrerão, que deixar de observá-lo”, e ela consistia em “não comerem caça, ou peixe, marisco, e outras muitas coisas” (Pereira, 1939, v.1, p.133-134). Ao estabelecer a relação entre a quijila e os Dez Mandamentos, ele ecoava o sentido que o termo era empregado nos catecismos em kimbundu. À luz da doutrina católica, condenou a quijila como um pecado e a definiu como “um pacto explícito, que fazem estes Gentios com o diabo”, demonizando-a, como a qualquer traço dos ritos africanos que identificou nas senzalas mineiras. Por esse pacto, “se assenta alguma conveniência corporal por parte do que o faz: como terem bom sucesso na guerra, fortuna na caçada, na lavoura etc.” (Pereira, 1939, v.1, p.134).

Observa-se que novo significado é atribuído à quijila, e sua nova função revela que passara a se encaixar no “complexo fortuna-infortúnio”, próprio das religiões dos sudaneses da Costa da Mina, especialmente das do Daomé. Esse enquadramento era uma reinvenção que resultava, “em primeira instância”, da “necessidade [de] enfrentar o infortúnio ou os ‘tempos de experiência difícil’, dos quais a escravidão é sem dúvida um dos casos mais extremos” (Parés, 2007, p.109-110). Não foi difícil para sudaneses e bantos amalgamarem suas culturas, pois, como dito, um processo de crioulização já ocorria na África, não sendo invenção puramente americana. Se no Ndodongo, em Matamba e no Congo, houve a fusão de elementos das culturas dos jagas, dos ambundos e dos kimbundos, da mesma forma, no Daomé, seus reis “após a conquista dos reinos de Alladá e Uidá, na década de 1720, ... adotaram uma política de apropriação dos cultos dos povos submetidos”, como estratégia para consolidar seu domínio (Parés, 2007, p.107), conquistas importantes para consolidar seu papel no tráfico de escravos. A novidade, como revelam as mudanças no sentido da quijila, era que a fusão passou a incorporar as crenças dos oriundos da Costa da Mina, que, vivendo apartados no continente africano, foram postos em contato no cativeiro americano, construindo pontes entre suas culturas de origem. Foi nesse novo contexto da escravidão, desterrados de suas terras, que diferentes grupos vindos do centro e do norte da África “tornavam-se verdadeiramente ‘africanos’” (Sweet, 2007, p.142), noção inexistente em seu continente de origem.

Pereira (1939, v.1, p.134) esclarece que “esta Quijila, ou pacto, passa por tradição a filhos, netos e mais descendentes”, do que se observa que, no contexto americano, a transmissão ambundo pela linhagem foi revivida, pois contar com a presença dos ngangas era mais difícil. Nas Minas Gerais setecentista, sob o impacto e moldada pela experiência do cativeiro, a quijila agregou elementos do rito original jaga (uma das ijila prescrevia tabus alimentares); da cultura ambundo, estruturada na linhagem; e das religiões daometanas; baseadas no complexo fortuna-infortúnio.^2^ Não foi só isso, porém; nesse jogo de constante reinvenção, na capitania, a quijila acabou por se transformar numa doença, com sintomática clara e perfil de doentes restrito.

## A quijila como doença

Segundo Antonio Cialli (16 jun. 1749, n.29, f.6v), as quijilas “eram uma queixa verdadeiramente horrorosa e incurável, frequentíssima nos Negros ..., sendo as verdadeiras chagas cancrosas [*sic*], ditas Fagederas”, isto é, feridas que apresentavam a carne morta ou úlceras corrosivas. Justificou que não se estenderia sobre o tema porque “discutirei em um tratado que tenho entre mãos das queixas endêmicas e próprias da América” (Cialli [Romano], c.out. 1749, f.3-3v), mas, infelizmente, esse escrito encontra-se perdido.

O primeiro caso que descreveu, em 1749, acometeu “um negro, por nome Antonio, escravo de Manoel Neto Covas”, que estava “cheio de chagas por todo o corpo”. Ao chegar na fazenda de Filipe Rodrigues, a mais próxima da Lagoa Grande, situada em Minas Gerais, em 1742, “lavando-se [com suas águas] algumas vezes no decurso de dois meses, se achou são” ([Cialli], 1749, p.8). Ele foi encontrado por um mulato forro, que, “indo este um dia à caça por divertir-se, e chegando ao caminho do Rio das Velhas, reparou gemendo entre as ervas, pouco afastado da estrada, um negro”. Era Antonio, já expulso, por seu senhor, da “fazenda e cativeiro, por incapaz para o trabalho em razão de uma queixa ... chamada vulgarmente = quigila” (escrita com “g”) (Cialli [Romano], c.out. 1749, f.3). Eram terríveis as condições que os escravos enfrentavam sob o cativeiro: o trabalho os fazia doentes e os incapacitava, e, uma vez sem condições de trabalhar, seus senhores deles se livravam, impossibilitando sua sobrevivência.

Em seguida, “movido o mulato [forro] da caridade cristã, conduziu, no seu Cavalo, o Negro à fazenda mais próxima, mais para lembrar-lhe o nome de Jesus do que para curá-lo”. Eram as terras de Filipe Rodrigues, onde o colocaram “em uma casa em que se costumava guardar o milho” mas, “como fosse inaturável o fétido das chagas, o mandaram lavar-se a miúdo no Lago, em cujas águas, que [se] achava[m] tépidas, não duvidava deter-se por dilatado espaço o enfermo” (Cialli [Romano], c.out. 1749, f.3). Para surpresa de todos, com “poucas semanas contava de banhos, quando repararam que mais desembaraçado passeava, e que as chagas tinham já outra aparência, até que admiraram finalmente indo as melhoras em conhecido aumento, estar em poucos meses de todo livre, e extinta tão horrorosa queixa” (Cialli [Romano], c.out. 1749, f.3).

Depois que frei Antonio de Miranda passou pela fazenda, as novas sobre as virtudes curativas das águas da Lagoa Grande começaram a se espalhar. No final de fevereiro de 1749, o religioso relatou, em Sabará, entre outras, a cura da quijila no escravo Antonio, e a notícia voou entre os plantéis de escravos das redondezas. Ao chegar à Lagoa, em 19 de março, Cialli (16 jun. 1749, n.25, f.6) lá encontrou alguns doentes atacados do mal já banhando-se e revelou que eles estavam “com dedos já consumptos, ocularmente cicatrizando”, revelando que os que buscaram a lagoa para se curar do mal eram mais numerosos, mas em seus manuscritos apontou apenas os primeiros que se curaram. Saliente-se que um número significativo de escravos oriundos da Real Extração dos Diamantes foi enviado do Tejuco, pelo médico do hospital local, para se banhar em suas águas, mas não é possível saber as doenças que lhes acometiam (Furtado, 2022a, p.135).

Em maio, além de Antonio, mais cinco doentes de quijila apresentavam melhoras acentuadas. Eram eles (1) Antonio, escravo do tenente Manoel Teixeira Lomba, que tinha as mãos e os pés aleijados devido à enfermidade, mas que, depois dos banhos, já foi capaz de andar; (2) o preto forro Francisco Xavier Barreto, morador no Funil, junto ao rio das Velhas, que estava “com as mãos aleijadas” e que, “com alguns banhos, estão quase naturalmente desfeitas”; (3) Jorge, escravo de Jacinto de Sá, que se encontrava imprestável por ter-lhe o mal atacado os pés e comido os dedos, razão pela qual seu senhor também o havia expulsado de casa, mas que “com um mês de banhos se vêm as chagas cicatrizadas, e quase todo fechadas”; (4) Paulo, escravo de Antônio Carlos Moreira, morador na vila real de Sabará, que sofria da doença há sete anos, e estava “com princípio de quigila na perna esquerda”, estando a metade inferior, junto à tíbia, bem inchada. A enfermidade, “a modo de cupim”, atacara-lhe um pé, que, coberto de feridas, quase se separara da perna, mas 17 dias de banhos foram suficientes para ela desinchar e voltar “quase [ao] natural”; (5) Manoel, escravo de Manoel Rodrigues, que havia 17 anos estava no Brasil, sofrendo da doença há trinta anos, iniciada ainda na África. Primeiro arrebentaram-lhe os metatarsos, depois caíram-lhe alguns dedos, ficando o restante muito inchado. Depois de tomar banhos “há mês e meio esta[vam] fechada[s] mais” da metade das chagas ([Cialli], 1749, p.12, 13, 20-21).

A partir dessas descrições, pode-se perceber que, em Minas Gerais, a quijila transformara-se numa doença que atacava apenas os negros, não importando sua origem, se do continente africano ou do Brasil. Apesar de mulheres negras não terem sido listadas entre os doentes, elas certamente não estavam imunes ao mal. Seus sintomas eram inchaços dos membros – braços e pernas –, aparecimento de feridas que não cicatrizavam nas extremidades – mãos e pés – e, em estágio mais avançado, perda dos dedos dos pés e das mãos. Cialli não descreve a origem do mal, mas o dicionário de português, do brasileiro Antônio de Morais e Silva, publicado em 1789, ajuda a sanar algumas lacunas, revelando que, no Brasil, transformações continuavam a ser operadas na quijila. Seu dicionário não apenas atualizou o de Bluteau, que lhe serviu de base, como incorporou vários termos correntes ou surgidos no Brasil, explicando seus significados.

No verbete, a “quigila” (com “g”, como Bluteau e Cialli escreveram) é definida como a “antipatia, que os pretos de África têm com alguns comeres, ou ações, de sorte que, se os contrariam nisso, padecem doenças, e talvez lhes segue a morte”. Novamente não há referência de que a doença estivesse circunscrita apenas aos nativos de Angola, como também não distingue o local onde ela se disseminava, podendo ser a África, o Novo Mundo e mesmo Portugal, para onde eram destinados os escravos africanos. Também não são especificados quais alimentos eram interditados, e afirma que “dizem alguns que estas antipatias de seus pais que os se contravêm a elas, vindo do outro mundo a isso as suas almas” (Morais e Silva, 1789, p.277). Reverberando seu significado original, suas transformações operadas na África e juntando o que Cialli observara, a quijila se tornara tanto a interdição alimentar como uma doença resultante da quebra do tabu. O papel dos pais continua determinante, dispensando a intermediação dos sacerdotes africanos, mais difíceis de ser encontrados ou de manter suas funções no contexto do escravismo colonial. Agora, porém, os pais aparecem na forma de almas de outro mundo, refletindo também a quase impossibilidade de o africano manter sua unidade familiar uma vez atravessado o Atlântico como escravo. Esse papel dos mortos era central na cosmologia dos grupos da África centro-ocidental, que compreendiam o universo como sendo dividido entre o mundo dos vivos e o dos mortos, mas existindo fluidez entre eles. Os parentes mortos continuavam a vigiar e a interferir na vida dos seus familiares vivos, cabendo-lhes inclusive impor obrigações sociais e morais e, “por vezes, os espíritos dos antepassados invocavam doenças como forma de castigar aqueles que tinham falhado com suas obrigações familiares” (Sweet, 2007, p.128-129). Observa-se que o tráfico atlântico e a escravidão conformavam as transformações operadas na quijila em fins do século XVIII. A quijila transformara-se numa “doença-punição”, pois passa a ser “a consequência necessária do que o próprio indivíduo ou o próprio grupo provocou”, uma “sanção que resulta diretamente da transgressão de uma lei”, no caso, os tabus alimentares prescritos desde a África (Laplantine, 1991, p.228-229).

A novidade que Cialli e Morais e Silva revelam é que, no Brasil, a quijila que se transformara numa doença e, ainda que atacasse só os negros, teve sua crença compartilhada entre senhores e escravos, os primeiros exemplificados no médico e no dicionarista, e os segundos nos negros que buscaram a cura na lagoa. No verbete, sob o espírito iluminista racional, a quijila foi enquadrada no sistema de simpatias e de antipatias, característico da medicina hipocrática-galênica, inserindo-a, da mesma forma que fizera Cialli, nas bases epistemológicas sob as quais se estruturava o conhecimento europeu. A cultura ocidental se apropriava, mas ao mesmo tempo modificava, a afro-americana que se formava no Novo Mundo, para ajustá-la à sua maneira de compreender o mundo, o que pode ser entendido como um processo que denomino “intertradução”. Esse conceito parte do princípio de que transformações são operadas enquanto as ideias (o conhecimento) circulam, independentemente de onde partam e de onde cheguem, quando são intertraduzidas e modificadas, sendo moldadas aos condicionamentos locais. Ao se adaptar às novas culturas, as mesmas ideias voltam a se irradiar, chegando inclusive até os locais de onde partiram, modificando, por sua vez, a própria cultura que as havia forjado. É o que se observa com o conceito de quijila, que, sob o impacto do expansionismo português e do escravismo colonial, mudava tanto na África quanto no Brasil, sofrendo transformações dos dois lados do Atlântico, amalgamando-se como uma cultura afro-americana original que, não é de estranhar, também incorporava elementos europeus e era compartilhada pelos escravos africanos.

## A cura da quijila: um saber intertraduzido

A crença no poder das águas da Lagoa Grande na cura da quijila, compartilhada pelos negros que para ali se dirigiram, seus senhores que permitiram seu deslocamento e os médicos que recomendaram os banhos, também é exemplar do processo de intertradução que caracteriza essa nova cultura afro-americana.

No universo cultural banto, “a água era o elemento que dividia o mundo dos vivos, negros, do mundo dos mortos, brancos” (Souza, 2002, p.148). E, segundo Cavazzi (citado em Leguzzano, 1965, v.2, p.214), “no passado, os reis de Angola adoravam um tal ‘calunga’, que quer dizer ‘mar’ ou ‘grande senhor’”. Acreditava-se no poder curativo da água, e um dos sacerdotes ambundos, o “*Ntinu-a-maza*, que quer dizer ‘rei da água’, ... esconde seus amuletos debaixo da corrente de um rio” (p.96), incorporando suas virtudes benéficas. Os Jagas também adoravam a água, e Cavazzi se ressente que essa adoração se “manifestava pela veneração que demonstravam pelos rios e pelas lagoas” (p.215). Descreve que, “logo que descobrem água de longe, prostram-se no chão para adorá-la, endereçam-lhe orações, oblações e votos para não serem incomodados por ela, para sofrerem penas, para não sucumbirem às doenças e para serem socorridos nas suas dificuldades” (p.215). Entre seus locais sagrados, destaca a grande lagoa de Saxia, situada no alto rio Cuanza, no extremo oeste de Angola, junto ao reino de Malemba, onde Cavazzi viu com seus próprios olhos esses ritos de adoração serem praticados entre as populações locais (p.215).

Também segundo a medicina hipocrática-galênica europeia, a água era considerada elemento com forte poder de cura, sendo parte fundamental dos corpos humanos, usada como salutífera para todos os órgãos. Essa tradição remontava à medicina ocidental praticada desde a Antiguidade, sendo famosas as termas romanas. As ideias de simpatia e antipatia entre os quatro elementos de que se compunham a natureza e os corpos viventes eram aspectos fundamentais dessa teoria, e a crença no poder curativo da água ocorria por via simpática, pelos efeitos do vapor da água fervendo no corpo humano, que restauravam a saúde e combatiam as doenças. Em Portugal e em Minas Gerais, várias fontes de água medicinal foram descobertas no século XVIII (Furtado, 2019, p.76-83), visto que as teorias médicas europeias vigentes, entre elas o curismo, o termalismo e a hidroterapia, propugnavam o valor curativo da água. A farmacopeia de Chernoviz, que alcançou grande circulação no Brasil no século XIX, em extenso verbete, identifica as águas minerais do Brasil, de Portugal e da Europa, classificando-as em tipos, conforme os elementos curativos nelas presentes. As da Lagoa Grande, já chamada de Lagoa Santa, foram classificadas como “águas simplesmente termais”, sendo apontado que “se conservam sempre tépidas” (Chernoviz, 1996, v.1, p.274-290), conforme observou Cialli em seus escritos.

Observa-se que a crença no poder reparador da água era compartilhada pelas culturas europeias e africanas, o que explica por que senhores e escravos, oriundos de Minas Gerais e de outras capitanias (havia doentes da Bahia, de São Paulo, de Goiás e mesmo do Pará), buscaram os banhos curativos na Lagoa Grande e, num processo de intertradução, redefiniram de maneira conjunta a compreensão e a forma de cura da quijila. Para os europeus, mais que nenhuma outra, a lagoa simbolizava a prodigalidade com que a divina providência abençoara suas águas. Para os africanos, era uma das lagoas onde seus deuses “se abrigaram em suas águas” (citado em Leguzzano, 1965, v.2, p.215). Se as almas de seus antepassados e suas maldições eram capazes de atravessar a Calunga Grande, como denominavam o Atlântico, provocando doenças, também o podiam seus deuses e suas crenças, que protegiam seus corpos já fustigados pela dureza da escravidão.

## Considerações finais

Na *Gazeta Médica da Bahia,* de 25 de setembro de 1866, o médico Julio Rodrigues de Moura, atuante no Rio de Janeiro, numa carta ao editor, considerou a quijila (com “j”), que para ele era apenas seu “nome vulgar”, uma moléstia de atrofia muscular progressiva, associando-a à elefantíase-dos-gregos pelos seus “sintomas característicos”, entre os quais se encontrava a “anestesia cutânea” (Moura, 25 set. 1866, p.68-70). Para tanto, apoiou-se no depoimento do médico Antônio José Alves, professor da Faculdade de Medicina da Bahia, mais conhecido como pai do poeta Castro Alves (Costa e Silva, 2006, p.9-15), que estaria realizando um estudo sobre a doença.^3^ Alves apontara que a moléstia começava com o aparecimento de manchas esbranquiçadas, “em cuja área os tecidos atrofiam-se lentamente”, ficando a “pele insensível”. Em “marcha lenta, termina comprometendo o aparelho respiratório e o tubo gastro-intestinal”. As “complicações” a ela associadas, especialmente “as ulcerações da laringe, as diarréias e a pneumonia”, ao fim, “sucumbem os infelizes afetados”. Alves identificou, por sua vez, a quijila como sendo uma elefantíase, apontando que “é muito frequente na Bahia, onde ataca principalmente a raça negra. E onde é conhecida debaixo do [seu] nome africano” (Moura, 25 set. 1866, p.69). Pelo menos a raiz africana da palavra era lembrada! Ainda que Alves reconhecesse que era mais recorrente nos que identificou como de “raça negra”, escapou ao médico baiano que a razão disso residia na cultura de origem dos afetados, em que a quijila encontra seu significado, mais preocupado que estava em enquadrar a doença no quadro nosológico da medicina erudita ocidental.

Ainda que escape ao recorte cronológico deste artigo, não deixa de ser instigante apontar que a sétima edição do dicionário de Morais e Silva, de 1878, “melhorada e aumentada com o grande número de termos novos usados no Brasil”, além do significado corrente na edição de 1789, acrescenta que o substantivo “quigila” (com “g”) já era empregado com o sentido genérico, “familiar”, de “antipatia, aversão”, e registra ainda que era “vocábulo da língua ambunda” (Morais e Silva, 1878, p.540). Mas, em outros dicionários da época, não é nem mesmo lembrada a africanidade do termo. O enquadramento da quijila na medicina erudita ocidental, aos poucos, modificou seu significado, apagando as raízes centro-africanas do mal, pois, desde Hipócrates, os sintomas é que fundamentavam o quadro nosológico dos males, e não o lugar ou a cultura de origem dos doentes. Com o passar do tempo, o termo nem mesmo se refere mais a uma doença, a palavra apenas ressoando o sentido original de tabu ou interdição. O dicionário de Luís Maria da Silva Pinto, publicado em Ouro Preto, em 1832, já definira a “quigilha” (com “g” e “lh”), genericamente, como “o mesmo que antipatia” (Silva Pinto, 1832, p.quig). E no de Caldas Aulete, publicado em Lisboa, em 1881, é registrado “quisillia”, como “antipatia, inimizade, zanga ... rixa, pendência”, sentimentos que são devotados a alguém; e como “aborrecimento, impaciência, mal-estar”, que se referem a uma coisa ou a uma situação: “isto faz-me quisillia”. Revelam que surgira ainda o verbo “quisillar”, significando “causar quisillia a, zangar, incomodar, aborrecer ... enraivecer-se” e o adjetivo “quisillento”, significando “propenso a à quisillia, que causa quisillia” (Caldas Aulete, 1881, p.1458).

Entre os séculos XVI e XIX, a quijila se transformou entre a África e o Brasil, condicionada às dinâmicas internas da África, ao avanço dos europeus no continente e, por fim, às dinâmicas do tráfico de escravos e às agruras da escravidão na América. Num diálogo intertraduzido de lembranças e esquecimentos, nos dois lados do Atlântico, europeus, brasileiros e africanos compartilharam e forjaram uma nova cultura, e a quijila é exemplar dos limites, possibilidades e impossibilidades do seu diálogo cultural.

## NOTAS

1 Assim que percorreram toda o litoral da África, já no século XV, os portugueses identificaram três grandes complexos socioculturais que usaram para diferenciar as populações com que entraram em contato no litoral da África Negra, divisão que é transportada para os mapas do continente. O primeiro coincide com a Costa da Mina; o segundo com o complexo Congo-Angola-Benguela, estendendo-se até a Costa Leste, na região denominada Costa da Cafraria; e o terceiro com a costa norte-oriental, ocupada pelas populações Swahili, entre as quais era forte a origem e influência árabe (Furtado, 2021, p.131-147). Mais modernamente, os estudos etnolinguísticos identificaram os dois primeiros grupos, como sudaneses e bantos, e, ainda que possa se fazer críticas a essa classificação, que é exógena e produzida do ponto de vista dos europeus, ela continua operacional na historiografia.

2 Cabe destacar aqui, mas sua análise foge ao escopo deste artigo, que em cultos afro-brasileiros, que mesclam elementos religiosos de diferentes partes da África, ainda hoje, prevalecem interdições alimentares denominadas quizilas. Nesse caso, ver Quizilas... (7 mar. 2017).

3 Saliente-se que sua segunda esposa havia sido casada em primeiras núpcias com um traficante de escravos na Bahia, e, durante o matrimônio com Antônio José Alves, continuou a operar os negócios negreiros do primeiro marido, mesmo depois da proibição internacional do tráfico. Por essa razão, “que não cause estranheza o fato de os ganhos com o comércio de escravos terem contribuído para o bem estar da família Alves” (Costa e Silva, 2006, p.22).
