# Supplementary material for: Doenças de africanos, doença africana: transformações da quijila, entre a África centro-ocidental e as Minas Gerais, Brasil, séculos XVII e XVIII
Source: Hist Cienc Saude Manguinhos. 2023 Oct 23;30:e2023052. [Article in Portuguese] doi: 10.1590/S0104-59702023000100052 (PMC10593376; doi:10.1590/S0104-59702023000100052)
Supplement: Translation

# Translation: Diseases of Africans, an African disease: transformations of quijila between Central West Africa and Minas Gerais, in Brazil, in the seventeenth and eighteenth centuries

**DOI:** 10.1590/S0104-59702023000100052en

**Authors:** Junia Ferreira Furtado

**Affiliations:** i Brasil Professor, Programa de Pós-graduação em História/Universidade Federal de Minas Gerais and Universidade Federal de Ouro Preto. Belo Horizonte – MG – Brasil juniaf@ufmg.br

**Keywords:** Quijila, Disease, Africa, Minas Gerais, Slavery

## Abstract

This article discusses the origin of quijila/kijila in Central West African culture, more particularly in the cultural universe of the Imbangala (Jaga) and the Ambundu and Kimbundu populations who lived in the Portuguese regions of Angola and the Congo in the seventeenth and eighteenth centuries. Following this, it investigates how the concept of quijila was structured, comprehended, and transformed, both in Africa, where it was basically a food prohibition, but whose applications and meanings varied; and in Brazil, to where it was transported in the 1700s, and where it transformed into a disease which attacked blacks, especially Africans of various origins, being framed as such in the Hippocratic-Galen universe characteristic of that time.

In 1749, an anonymous pamphlet was published in Lisbon, entitled *Prodigiosa lagoa descoberta nas Congonhas das Minas do Sabará* ([Cialli], 1749; Furtado, 2022a, p.113-148). The theme of this article is an instigating disease described in the pamphlet called quijila/kijila and its objective is to seek its origin Central West African culture, more particularly in the Bantu cultural universe, experienced by the Jaga, Ambundu, and Kimbundu populations in the Portuguese regions of Angola and the Congo. Between the seventeenth and eighteenth centuries, quijila was transformed in Africa, until in eighteenth century Brazil it came to be a disease which attacked Blacks, especially Africans of various origins, and was framed in the Hippocratic-Galenic universe characteristic of medicine at the time.

*Prodigiosa lagoa* reported the discovery of the curative power of the waters of lagoa Grande (now called lagoa Santa), located in the comarca of Sabará, in Minas Gerais captaincy, in Brazil, and the cures of various sick people who drank from or bathed in its waters. It ends with a long list of the first 109 patients who bathed there and whose sicknesses were cured or mitigated, indicating their names, addresses, “and the type of complaint” ([Cialli], 1749, p.12-27). To these cases another four can be added, cited in the historical account, amounting to a total 113 sick people.

The text sees steel and vitriol as the prodigious elements of the water. Vitriol, a mineral salt similar to glass, acted externally and was used as an antidote and in the healing of sores, including of the genitals. Its presence in the water cured “all cutaneous complaints,” as well as open sores, “tumors, hernias, warts” and pains. Quijila is included along with scabies, leprosy, morphea (leprosy), formigueiros (cutaneous wounds in the form of holes made by ants), erysipelas, and all types of sores ([Cialli], 1749, p.9; Cialli [Romano], c.out. 1749, f.6-6v, f.7), considered to be external diseases.

## Sick slaves, slave diseases

Among the 113 sick people identified in *Prodigiosa lagoa*, 88 were men (77.9% of the total) and 25 were women (22.1%). Overall, 50 were slaves and 13 were freed (forros), equivalent to 63 people of color, or 55.75% of those who were sick. Among these six were attacked by quijila. All diseases were described, classified, and framed through concepts in force in Hippocratic-Galenic medicine, though quijila was the only one which just attacked Black people (Nogueira, 2011, p.47), as all the others made no distinctions between whites and people of color. Due to the terrible living conditions and diets to which slaves were submitted, there were diseases which were more recurrent or acute among them. For example, Pedro, a slave of Luiz Cardoso, in Caeté, after bathing twice expelled “three black worms, each three spans long,” which had caused him “great pains in the stomach.” Maria, the slave of Francisco Fernandes Braga, in Sabará, had “papo for years,” as goiter was called. Work accidents, especially in mining, were inevitable and Pedro, the slave of Alexandre Teixeira, from Santa Luzia, had an open wound for an year, after “an enormous log” had fallen on his foot; while Ignácia, a slave belonging to Brites Correia, who lived in Sabará, appeared with a cough and a pain in her chest, “releasing some blood,” after “a clothes trough had fallen on her breasts four months previously” ([Cialli], 1749, p.13, 16, 18-19).

As it sheds light on the diseases which affected slaves and unveils their living conditions, the text of *Prodigiosa lagoa* is of great interest to the historiography of slavery, science and medicine. However, the study of quijilia represents a greater challenge, since it was not only an ailment recurrent among slaves, but also a disease whose origins could be traced from the cosmologies of the people labelled as Bantu,^1^ from Central West Africa, especially the Jaga, Ambundu, and Kimbundu populations.

Although many of the other sick people listed had symptoms similar to those that the Italian doctor Antonio Cialli, author of the pamphlet (Furtado, 2022b, p.79-112), diagnosed as quigila, (spelt with a “g” in the text) no white person suffered from this disease. Comparing the cases of Antonio, a slave of Manoel Teixeira Lombo, João de Araújo, son of the latter, and João da Costa Ferreira, is very revealing. In the first case the illness attacked his hands and feet, which were crippled and covered in wounds, resulting in the loss of some fingers. João de Araújo, a free white only 18 years old, was also covered in sores and his legs were crippled due to the damage caused by the wounds in which they were covered. The same happened with João da Costa Ferreira, another white boy, aged 13, son of Manuel Jorge da Costa Ferreira, a resident of the village of São Sebastião, located on the banks of rio das Velhas, who suffered “from two wounds on a leg” and had “his big toe on his right foot eaten by another” ([Cialli], 1749, p.12). Although the illnesses and symptoms were similar among all three, quijila was only diagnosed in the slave and is not mentioned at all for the two whites. It seems that the presence of wounds that did not heal and inflammations in the limbs, resulting in the loss of fingers or toes, was not determinant in the diagnosis of the disease, but rather the type of sick person, in other words the fact that they were Black.

## Quijila and the Jaga

Although Luso-Brazilian health professionals had sought since the sixteenth century to incorporate indigenous and African knowledge about native plants from the New World and their uses in medicine, incorporating them in materia medica, medical treatises at the time framed diseases, even those which affected only or mainly the autochthonous and enslaved populations in the New World, within the nosology of European Hippocratic-Galenic medicine (Furtado, 2007, p.127-151). However, that was not what occurred on the pages of *Prodigiosa lagoa*. In an unprecedented form, a disease – quijila –, whose meaning and origin occurs within African culture and beliefs, was incorporated in the European nosological framework and not vice-versa. While the symptoms were not, in themselves, capable of leading to its diagnosis, to understand what the disease involved it is necessary to look for its origin within the Central West African culture shared by those who suffered from the illness.

The three treatises which Antonio Cialli wrote about the cures he observed using the water of Lagoa Grande are unique and rare cases of texts from erudite medicine at the time, which refer to a disease of African origin ([Cialli], 1749; Cialli, 16 jun. 1749; Cialli [Romano], c.out. 1749). In this way, the study of the disease and its origins contribute to elucidate some of aspects of the culture of slaves in Minas Gerais, in Portuguese America, which in general were erased, due to the hegemonic nature of Europeans in Brazilian territory, especially in relation to its diseases and healing practices.

We will begin with the origin of the term. In Kimbundu, a language spoken by the Ambundu populations of Angola, the basic sense of kijila is prohibition or even taboo. The word appears very early in the catechisms translated to Kimbundu, such as that of Francesco Pacconio and António Couto, published in Lisbon in 1642 (Pacconio, Couto, 1642), being used to translate the taboos imposed by “idols.” In the 1645 edition, it is also used to refer to one of the Ten Commandments, revealed by God to Moses, replacing the more secular term “milongo,” which came to be employed only in relation to laws or decrees issued by the king. The semantic distinction in the meaning of the two terms is clear, kijila is circumscribed to rules imposed by the religious universe and milongo to the secular.

The word quijila or kijila appears directly linked to the customs of the group the Portuguese called the Jaga, but originally did not refer to a specific illness or disease. It was any of the 14 laws or prohibitions and rituals followed by this group who lived in the interior of Angola far from Luanda, who are identified in modern ethno-history as the Imbangala. These were accused of being bloodthirsty anthropophagic savages who lived by ravaging the nearby Ambundu populations (Miller, 1973, p.121-149; Souza, 2013, p.141, 2018, p.98). The identification and the origin of the Jaga in the historiography of Africa, based on the European chronicles of the seventeenth century, remains controversial. The term was used to identify savage nomadic warriors who made violent incursions at this time into the Congo and surrounding areas, terrorizing Europeans and Africans, and initially not forming a particular ethnic identity. It seems that Jaga derives from the word aka, which means “the other,” and was applied to various groups seen by the local people of an Ambundu origin allied to the Portuguese as robbers, raiders, or outsiders, and who had made war and mobility their way of life. Some European chroniclers from that time identified their origin in the Zimba and Mumbo, people from the East coast, who had moved to the west by this time, due to their mutual practice of anthropophagy and nomadism. Generally speaking, the Jaga came to be described in the European literature and sources as nomads who never constructed a cohesive State. They were anthropophagic, lived by raiding and making war on local Bantu peoples and resisted the penetration and colonization of the Portuguese. They were opposed to the more numerous Ambundu, who were sedentary, lived on agriculture and grazing, and whose socio-political organization was based on lineage, forming States, with the most important kingdom in the region being the Ndongo or Dongo (Miller, 1973, p.121-149; Thorton, 1978, p.223-227; Hilton, 1981, p.191-202; Pinto, 1999-2000, p.193-243; Souza, 2018; Furtado, 2021, p.442-455).

Since the socio-political cohesion of the Jaga was not based on lineage and because they had never developed a State, adhesion and belonging to the group were “marked by a series of rites and by the observation of specific rules” (Souza, 2018, p.121), called ijila (plural of quijila), a set of rules or prohibitions. Initially, many of the ijila “were related to the role of women” while “the majority of others were concerned with rituals for funerals, promotions, or changes in social position, and mechanisms to initiate small children in the quilombo.” Among the Ambundu, the kilombo was “a society of initiation or a circumcision camp, where boys were prepared for the status of adult” (Schwartz, 2001, p.249-255). The Jaga adopted this type of organization, basing their identity on individual valor and not on lineage, since they consisted of groups of different origins. In the eighteenth century, the term kilombo came to be used by the Portuguese to identify the capitals of native kingdoms, especially those of the Jaga, who resisted their advances in Central West Africa, while in Brazil at the end of the seventeenth century, becoming generalized the following century, it was used for communities of escaped slaves (Lara, s.d., 2021).

One of the great informants and disseminators of the customs and the barbarity of the Jaga was the Italian Capuchin priest Giovanni Antonio Cavazzi (de Montecuccolo). In 1687, after working as a missionary in Angola, he published *Istorica descrittione de tre regni Congo, Matamba et Angola.* According to him, quijila (using the name in the singular) was a set of laws which were initially established by the Imbangala queen Temba-Ndumba, “applauded and respected as a woman of extraordinary courage,” and “divided into domestic, religious, and civil laws” (cited by Leguzzano, 1965, v.2, p.177-179). The domestic ones prescribed “the observance of some ancestral traditions, such as abstinence from pork, elephant, snake, and some other animals,” although he asserts that “all of this implies a great violence to the natural appetite which everyone has,” due to the anthropophagy they practiced (cited by Leguzzano, 1965, v.2, p.179-181).

In a prejudicial manner, from a Catholic perspective, Cavazzi considers them “ridiculous and superstitious,” stating that these laws “have as their object some prescriptions which, from one moment to another, are invented by cunning and discerning sorcerers, according to the opportunity and genius of those wretches” (cited by Leguzzano, 1965, v.2, p.180). Sorcerers is how he pejoratively refers to the ngangas, or gangas, as African priests were called, who, according to him, “deal with private life, disputes, diseases, the dangers of death, and other similar subjects” (cited by Leguzzano, 1965, v.1, p.180). Quijila was in his perspective the negation of Christian principles and the symbol of the barbarity of the Jaga. It also echoed the view that the Ambundu had of these commandments, which they considered inhumane, since in Mbundu cosmology they were considered cruel.

From the Capuchin’s description, it can be seen that the alimentary prohibitions did not correspond to the entirety of the Ijila, but were part of the domestic quijila, imposed by priests and transmitted from ancestors by oral tradition. The prohibited animals were “pork, elephant, snake, and other animals” (cited by Leguzzano, 1965, v.2, p.179), however Cavazzi does not specify what penalties offenders suffered, nor that it caused a disease. The prohibition of the eating of certain animals, especially those of a large size, was not exclusive to African peoples, but recurrent in various cultures, including in seventeenth century Europe, in which it acquired various functions, especially the reservation of the meat of some animals, mainly of a large size, to privileged individuals, generally from a superior hierarchical position, such as kings (Thompson, 1987).

Ijila were first adopted by part of the Ambundu population during the reign of the famous queen Jinga or Njinga, who claimed for herself in 1624 the Ndongo throne. Fighting against the Portuguese, who had the support of some allied Jaga (who immediately abandoned ijila), she established an alliance in 1629 with the Imbangala chief Cassa, with whom she actually married, adopting the Jaga traditions and assuming chiefdom of the group. Under pressure from the war, in 1630 she established her kingdom in Matamba, west of the Cuanza river, from where she commanded resistance to the Portuguese until 1656, when she made a treaty with the Portuguese and reconverted to Catholicism, which she had professed during her youth.

Without ever totally abandoning lineage as a group identity, as she carried with her a funerary urn with the bones of her ancestors, Njinga also adopted the ijila precepts and prohibitions (Heywood, 2019, p.125-128, 197-227; Souza, 2018, p.110-114, 128-130). Furthermore, among all the local leaders she occupied a special role due to her resistance to the Portuguese in the interior among the Ndongo and Matamba, “starting to live according to the kijila laws and gathering around her those who resisted the Portuguese conquest,” whether they were Jaga or Ambundu. Actually, “being Jaga did not eliminate a possible Ambundu ethnic origin” (Souza, 2018, p.128, 152). In fact, it should be noted that for the colonizers Jaga and ijila were associated with anthropophagy, savagery, and barbarism, and were attributes conferred on the Queen and her subjects. It was by identifying with the former and the adoption of the latter which allowed Nijinga to attract all those who resisted the Portuguese, irrespective of their ethnic origin or language, which, in the 1640s, transformed Matamba into the largest African state in the region and contributed to the greater diffusion of ijila rites among the local population.

## Quijila and its creolization in Africa

In 1656, under pressure from the nonending war, Njinga made peace with the Portuguese, after resisting them for around 40 years. Among the conditions imposed on her was the abandonment of ijila, the adoption of Catholicism, and the conversion of her subjects. Italian capuchin missionaries were dispatched on evangelizing actions and sought to eradicate elements of African culture considered heretic and barbarians. Cavazzi (cited by Leguzzano, 1965, v.1, p.113) was one of the most important of these. In his writings the cleric warned that “these Blacks practice various superstitions,” which he “did not like to narrate, since they are ridiculous,” and apologized that he did so “only to instruct missionaries, so that they could take the Blacks away from their errors.” Among these errors, his greatest condemnation was aimed at the quijila which he attributed to the Jaga.

Even after the arrival of the Capuchins, Nijinga behaved in a paradoxical manner. On the one hand, she transformed “Matamba into a center for spreading Catholicism” (Souza, 2018, p.143), adopted the Christian name of Ana de Sousa and sought to restrict the action of the ngangas and the xinguilas – the latter were responsible for making connections with the world of the dead – trying to force her subjects to abandon African rites and customs. On the other hand, under the pressure of the elites and priests, Ambundu and Imbangala customs and rites, including ijila, were preserved, which were important sources of legitimation for the legitimation of her power. This is what can be observed in her funeral, witnessed by Cavazzi, who had to tolerate the alteration of Catholic, Ambundu, and Jaga rites. A tambo, a Jaga burial ceremony, was carried out, and the only thing that the cleric could prohibit was the practice of human sacrifice (cited by Leguzzano, 1965, v.2, p.156-157, 161; Souza, 2018, p.143, 160, 167, 170).

Under the pressure of the clerics, during her reign, “the kijila laws [lost] force, in favor of Ambunu rules” and “her people constructed new identities, resulting from the situations then being experienced”, which combined “traditional elements with the innovations introduced by the missionaries” (Souza, 2018, p.207-210). Following the death of the famous queen, the policies of her successors oscillated, tending to repress the practice of Catholicism and to return to African customs. In the burial ceremony of Njinga’s sister, baptized as d. Bárbara, who succeeded her, for example, the tambo was once again celebrated with human sacrifices (cited by Leguzzano, 1965, v.2, p.168), while in the reign of Jinga-Mona, who succeeded the latter, the missionaries were expelled. However, this did not involve only the reconstruction of an Ambundu identity, based on lineage. Like the tambo of d. Bárbara, various Jaga customs still remained, albeit transformed. Let us see what happened with quijila in this context of change.

In 1682, the Capuchin priest Girolamo Merolla da Sorrento arrived to act as a missionary in the region, 10 years later publishing the *Breve e Succinta Relatione del Viaggio nel Regno di Congo...* In a passage referring to the Congo, he refers to quijila, which he calls cheguilla, as being the sixth oath. However, the oaths which the Ambundu swore had another nature and distinct functions from the Ijila, as they were used to arbitrate justice and consisted of various trials to which the individual was submitted to prove their guilt or innocence (cited by Leguzzano, 1965, v.1, p.102-113; Ferreira, 2013, p.196-201). Here it is important to note that Merolla da Sorrento uses a cognate of the term, in Kikongo, spoken by the population of the Portuguese Congo/Kongo, but he attributed the meaning which quijila had in Kimbundu, even though it is probable that he was aware of the distinct ways the term was used in the two societies. According to him, in the oath, “as is the custom, the parents, or the sorcerers, create rules that the children have to inviolably obey,” which consisted of “abstaining from eating any type of chicken, or the flesh of wild creatures, or fruits of this type, or raw roots, or cooked in this manner, or another manner, with a bestial difference” (Sorrento, 1692, p.146-147). In other words, it was only related to alimentary prohibitions.

The priest, to whom everything seemed ridiculous, was astonished “that they would rather fast for some days that eat forbidden things,” since “they are sure they would die soon after.” It is important to highlight that for him, these precepts were dictated by the mother and only if she “does not give them Chegilla” then “they will go immediately to receive from the Wizards (Magi),” since “they are certain that they will die soon” (Sorrento, 1692, p.146-147). The reference to the ngangas as wizards or sorcerers reflects their dispute with Catholic missionaries over the power of communication with the supernatural. Following this, Sorrento narrates an exemplary case of “a Black man” who on a journey was served with a wild hen by his host. Despite being asked, the latter hid from him the information about the wild origin of the animal. Four years later, when meeting the host again and being told of the real origin of the hen, the guest concluded that it “had Chegilla,” and “he was so distraught that he did not manage to survive for more than twenty-four hours,” since for “these people, when they are sure that they have transgressed Chegilla, death comes quickly to them” (p.146-147).

Merolla da Sorrento’s description of quijila was different from that of Cavazzi in a number of ways: (1) it no longer refers to one of the rules of ijila but to an oath; (2) it was limited only to the prohibition of food; (3) the main transmission of prohibition is through the mother and only in her absence through the native priests; (4) the forbidden food is slightly different and prescriptions have been added referring to the preparation of food; (5) disregarding the prohibition would result in the death of the transgressor. Since it is known that Sorrento was an attentive reader of Cavazzi’s book, which also served as inspiration for his own, these differences could not be credited to his ignorance of the latter work or of Kiongo, or to difficulties of understanding what he had observed, but rather to the transformations which operated in the local culture under the impact of Catholicism and, especially, the progressive hegemony of Ambundu customs over the Jaga among the Angolan Bantu and even among the Congolese, since cultural exchanges among these peoples were frequent. The missionaries were implacable towards human sacrifice, which was an important part of ijila, and they persecuted and sought to delegitimate the power of African religious leaders.

Various historians of Africa use the concept of creolization (Mintz, Price, 1976, 2003) as a theoretical tool to understand the transformations operated in Central West Africa under the impact of Christianity and the socio-political institutions introduced by the Portuguese, although they do not always agree on its meaning. According to James Sweet (2007), the concept does not make sense for societies from the interior of the continent that were not impacted by the Atlantic traffic. While for Linda Heywood (2008), amongst others, its use is valid both for the coast and the interior, since its analysis also centers on miscegenation between Portuguese and African culture (Ferreira, 2013; Schleumer, 2018; Lara, 2021, p.140-152), theoretical conceptions which can be instrumentalized for the study of the transformations which quijila underwent in the Angola-Congo complex.

However, the transformations operated in Central West Africa in a short period of time in Africa can only in part be credited to the action of Catholic missionaries, who could be condescending towards alimentary restrictions, but not to the human sacrifices of the Jaga’s ijila. The fact that the main transmission link was transferred, as Merolla da Sorrento certifies, from the nganga to the mother did not occur only due to the impact of Christian clerics. Rather it is also a reflection of the progressive hegemony of Ambundu cosmology in the Jaga-Ambundu communities, which brought together those who resisted the Portuguese in the interior of Angola. For the Ambundu, “the cult of ancestrality had a specific role in articulating social relations within the kinship group (lineages)” (Marcussi, 2015, p.153) and they had reservations about the inhumane aspects of the Jaga sacrifices as well. In turn, the presence of the concept of quijila among the Kimbundu in the Congo can be explained by the cultural exchanges which took places among the populations of Central West Africa, which intensified after the arrival of the Portuguese. The term creolization can be used to analyze these transformations, but in a broader sense. It helps to elucidate the impact of the Portuguese, Christianity, and the slave trade, and the incorporation of the cultural practices of different local groups, the case of the Jaga, the Ambundu, and the Kimbundu, which were amalgamated and transformed, responding to the daily anxieties imposed on their native societies, specially by the Europeans.

## Quijila and the cure of quibuco

The undated and anonymous Portuguese manuscript “Ritos gentílicos e supertições que observam os negros do gentio desse reino de Angola desde o seu nascimento até a morte” (Gentile rites and superstitions observed by the gentile blacks of this kingdom of Angola from birth to death) provides other clues about the creolization process which, still in West Central Africa, operated within the concept of quijila. Amongst other topics, it describes the infirmities and healing rites used in Angola which, as can be observed from the title, is described from the anonymous author’s point of view, who always sees them negatively, pejoratively classifying them as superstitions and gentilic rites, in other words, pagan or idolatrous.

What calls attention in the text is that the role of the ngangas is said to be restricted to the healing of illness, while they are described as a mixture of sorcerers and surgeons. The latter is the term which the author of the manuscript finds in European culture to identify to them by analogy. It is evident to the European reader that, as surgeons, they had practical knowledge, though they were badly educated and ill-prepared, which implied their disqualification in relation to doctors, the only ones who had erudite knowledge. The use of the term surgeon and the way their role is described obliterates the important religious roles and the part they played in the transmission of the customs and rites of the ancestors which the nganga still performed in Africa. In the text, their action was circumscribed to looking after the sick and to prescribing the customary treatment since, due to the pressure of the Portuguese, in spiritual questions they were increasingly replaced by Catholic priests or had to camouflage their spiritual actions as curative practices.

According to *Ritos gentílicos...* (s.d., f.2), “when some [individual] seems sick with a lesion, in their understanding they said he has quilundos, [and] for those who cure themselves they consult a surgeon called nganga de quilundos, ... and the so-called surgeon goes to another room without anyone, where they invoke the devil, whom he consults about the illness.” Once “health is achieved, the quilunfo is celebrated, which is the idol invoked with much comiraina [*sic*, food] in thanksgiving.” For the anonymous author, popular medicine and sorcery were consubstantiated in the practice of the ngangas, who invoked for healing not only their idols, but the devil in person, which signified the reading of their rites and their beliefs from a Catholic point of view, condemning them, since idolatry and demonology marked their practices of cure. Africans were seen as others, and their religion as something of the devil (Schleumer, 2018).

Among the infirmities which the text describes, “there is another disease which is the illness of quibuco, for the cure of which the quibuco idol is consulted,” without explaining which symptoms and part of the body is affected. In the manuscript quibuco means both the idol which is consulted and the illness for which a cure is sought, which was one of the “ailment[s] of imagination” which Black people suffer from (Ritos gentílicos…, s.d., f.2). The treatment consisted of the sacrifice of goats and chickens, offered to the idol, whom the ngangas invoked. Once health is recovered, “the surgeon … gave those cured their precepts of eating or not eating this or that, precepts which were called Quigilles, which they strictly observe” (f.2). Quijila continued to refer to the “precepts of eating or not eating this or that,” but which now are restricted to the period of convalescence of a specific disease, quibuco, and prescribed by the nganga, recovering in part the latter’s role as intermediary with the supernatural, but restricted to the universe of healing diseases.

Although the text is not dated, it can be seen that new transformations were taking place. Disconnected from the ijila, of which only the name was preserved, quijila was no longer a rule, nor a broad set of food prohibitions, but had become restricted to an alimentary diet, prescribed by the ngangas, though only after the curing of the illness of quibuco. For the first time, quijila is associated with a specific disease, although it is not the disease in itself, but the consequences of breaking the diet are not described. It is thus not exactly a taboo.

Above all, this description of quijila reveals that, over time, it became a rite observed by all the “gentile Blacks in this kingdom of Angola,” no longer being restricted to the Jaga, nor circumscribed to the Matamba kingdom. Stripped of its original meaning, it spread through the Ambundu populations in the region of Portuguese Angola. This expansion reveals once again the creolization which the different cosmologies of the local Bantu peoples underwent, as they exchanged rites and beliefs. A key to understanding this consubstantiation and its dissemination is Cavazzi’s affirmation that after the deaths of Nzinga and d. Bárbara, her successor Jinga-Mona invoked through the xinguilas the spirit of the former in a public square, re-enacting the importance of lineage to structure his power. However, after this, “he ordered that all the prisoners from the previous expedition be sacrificed” and “and that the stomachs of many of those present be cut open and both he and the soldiers be sprinkled with their blood, making happy wishes for their prosperity” (cited by Leguzzano, 1965, v.2, p.170). This was a restaging of the most important quijila of the Jaga, the one instituted by Queen Temba-Ndumba, which was followed by her successors, including Nijinga, which consisted of anointing the body before a war with a magical ointment, the principal ingredient of which was the bodies of the sacrificed children. It can be noted that Jinga-Mona legitimated his power by staging important rites for the Ambundu and Jaga and after the ceremony, he sent “messengers all over the kingdom that ... the old ceremonies be renewed and that each person could live freely according to the Jaga rites ... and without any impediment at all reintroduced the old rites” (cited by Leguzzano, 1965, v.2, p.170).

## Quijila as a curse

At the beginning of the eighteenth century, according to Rafael Bluteau’s Portuguese dictionary, published in Lisbon in 1720, quigila (written with a “g”) came to be seen as a “curse, which the parents of the Blacks of Angola tell their children, saying that if they eat venison, lamb etc., it will curse them, and they say that if they eat it, they will see spots or other marks, and die” (Bluteau, 1720, p.58). Changes in its meaning occurred once again, or at least in the understanding that Europeans, particularly the Portuguese, had of African quijila. Food prohibitions, present in the original taboo, continued to be mentioned, though the term does not actually refer to it, but to the curse caused by the breaking of the diet, although Bluteau does not refer to the curse as a specific disease.

The term curse circumscribes the comprehension of African culture as sorcery, as was typical of Europeans, which echoed the intolerance of Catholic missionaries to African rites and beliefs. On the part of the Church, there was a model in which “masters and slaves, whites and Blacks had to be Christians above all ... In this model, there was no tolerance of practices of an African origin, seen as demonizing” (Souza, 2002, p.229). The vision of the continent is then marked by the negative, by intolerance in relation to the habits, religions and rites, and customs of the land, amongst others, while alterity is what marks the relationship of Europeans with Africa.

Bluteau (1716, p.734) describes as a consequence of disobedience the appearance of “spots (nódoas), other marks, and death.” Nódoas were, according to the same dictionary, spots which appeared mainly on the face. It can be noted that, despite the fatal destiny awaiting the transgressor, nódoas are less symptoms of a disease and more marks and public signs, imposed by parents, which leave clear for the community the transgression practiced by their children. This understanding explains why for Cialli, “the black nódoas,” which appeared “on the face, arm, and legs” of Francisco Moura Chagas, in which “he gradually lost feeling,” were not diagnosed as quijila. The latter was white and free, an inverse profile of those affected by quijila, and it was believed to be a consequence of the fact that, “three years previously after getting wet, he had pains in his hand and right foot, from which he immediately lost feeling on the outside part” ([Cialli], 1749, p.23).

According to Bluteau, at the beginning of the eighteenth century, the belief in quijila was shared by all Africans in Angola, without distinguishing the group to which they belonged, and incorporated elements of Jaga culture, the case of the food restrictions, and also of the Ambundu, with the importance given to lineage – the curse came to be imposed by the parents and not only by the mother, as had appeared among the Kimbundu. In this sense, he returned to Merolla da Sorrento’s information that the intermediation of native priests was no longer necessary, revealing the decline in their power in light of the advance of Catholicism in Africa, but also in the Americas, where slaves faced numerous difficulties to transplant their cults intact, one of which was the absence of their religious leaders. Nor is there any mention of it being a diet followed during a period of convalescence, or any relationship with a cult in particular, the case of quibuco, as has been described in *Ritos gentílicos...* Also missing is an explanation of the meaning and the reason for these prohibitions, or why they are limited to only two animals – deer and sheep. Perhaps the dictionarist did not know. The choice of these animals seems to have arisen out of disputes in Europe between kings and subjects and in the Americas between masters and slaves for large game hunted in the forests and the need to preserve the herds of the firsts in light of the daily hunger of the slaves. In this sense, the mention of sheep and deer and the transmission of the curse by parents and not only by the mother added a further ingredient to the creolization of quijila, the Atlantic slave trade and the massive importing of slaves into Portuguese America, which brought with it a repertoire of beliefs and customs already being transformed in West Central Africa and which, after crossing the Atlantic, suffered new pressure for change.

## Between Angola and Minas Gerais, a brief interval

The wars which the Portuguese waged in the interior of Angola during the seventeenth century against the Jaga and especially against the resistance led by Queen Njinga in Ndongo and Matamba, produced numerous slaves for the Atlantic trade. After 1649, the flow of slaves increased following the defeat of Njinga and the signing of the peace treaty, which reopened the markets of the interior of Angola, allowing the Portuguese to supply themselves with slaves. On this occasion, the queen sent a present to the governor, the bishop, and the main judge (ouvidor geral) of Luanda, consisting of “some heads of slaves from the runaway people,” who had lived in the kingdom for a long time, as well as the 2000 she sent as a ransom for her sister who was imprisoned in the capital (Souza, 2018, p.139-141, 155, 161). Due to the missionary action of the Capuchins, ngangas caught practicing the prohibited old Jaga rites were also sent to the Atlantic slave trade. According to Cavazzi, one of them “was sent with his two companions to the mines of Rio de Janeiro;” another, “with all his family,” was condemned “to the mines of the Americas” and a third “was whipped through the streets of the city [of Santa Maria de Matamba] and finally dispatched with other companions to the Americas” (cited by Leguzzano, 1965, v.2, p.145-146). The mines of Rio de Janeiro refers to Minas Gerais captaincy, in Brazil, where huge golden mines were found.

The war against those who resisted the Portuguese advance extended the “frontier of slavery” into the interior of Angola (Miller, 1988). Many of the recent arrivals in the Americas were sent to Minas Gerais captaincy, where the need for slave labor grew to the same extent as the area of gold and diamond mining. It is calculated that at the beginning of the eighteenth century, around 40% of the newly arrived slaves were sent to the mining area (Goulart, 1975, p.165), which on average represented around 1560 slaves per year (Ribeiro, 2005, p.195).

Furthermore, the colonization of Minas Gerais captaincy occurred at a moment of a change of direction in the African slave trade, until then dominated by the “Angolan wave” (Miller, 1988; Lara, 2021, p.185-199). From the end of the seventeenth century onwards, the region of Costa da Mina began to increasingly meet the demand. Mostly of Sudanese origin, in the captaincy they were generically called “minas.” In the two-year period of 1725-1727 alone “around 5,700 slaves entered the port of Rio de Janeiro annually, coming from Costa da Mina and Cabo Verde. Of these, 2,300 [were] afterwards transferred to Minas” (Soares, 2000, p.77). However, during the eighteen century the number of mina slaves declined, while Bantus, from the Congo-Angola-Benguela complex, began to rise in numbers. Calculations made for the 1720-1888 period, based on *post-mortem* inventories, showed that 28.3% of Africans were of a Benguela origin, 23.9% from Angola, 10.7% from the Congo, while only 10.5% were from Mina (Bergad, 1999, p.151).

Based on the profile of the composition of slave traffic and the observation that along with the slaves coming from Angola (and perhaps also from the Congo), their beliefs crossed the Atlantic, it is thus worth inquiring how knowledge of quijila was transmitted among slaves in Minas Gerais, what meaning it acquired among them, and if it spread to slaves with other origins. Studies of slavery in Brazil have increasingly incorporated the problematization of the ‘survival’ of African cultures in the Americas and the ‘adaptations’ which they underwent in the new context” (Lara, 2021, p.124). Mining demanded a numerous contingent of slaves, a heterogenous community which, within the senzala, promoted interactions and reappropriation among distinct cultures brought from their native lands, but who, in the context of slavery, were amalgamated and molded by it. As recent historiography has noted, “African culture was an important element in understanding the logic of slave actions, but the experience of slavery itself has to be taken into account” (Lara, 2021, p.125). In slavery, where Africans from a wide variety of origins lived with and exchanged beliefs and rites, there occurred a “reconstruction, reinvention, or reinstitutionalization of African religions in Brazil,” (Parés, 2007, p.109). This was possible because “the fundamental cosmological principles (explanation, prediction, and control) shared by the majority of these African peoples allowed them to develop common conceptions” (Sweet, 2007, p.142), even coming from different regions of the continent.

In accepting the occurrence of a process of Africanization in the Minas senzalas, it becomes less important to know the origins of those affected by quijila who bathed in the lake. Five were slaves and one was a freedman, though for only two of them is it certain that they are African: the Black freedman Francisco Xavier Barreto (case 12), since the designation of color, Black man or woman, was attributed in the captaincy to those brought in from Africa via the slave trade, in contrast with the creoles, as those born in Brazil were called; and Manoel, the slave of Manoel Rodrigues (case 75), since he had contracted the disease in Africa 17 years before coming to Brazil ([Cialli], 1749, p.13, 21). However, it is not possible to know for these two in what part of the continent they originated, whether they were from the Congo-Angola complex or Costa da Mina. The doctor Antonio Cialli refers to quijila as being a disease of Blacks and Black was how authorities in Minas Gerais usually referred to the slaves of African origin. While their number appears reduced (only six sick people), the appearance of the term quijila in the dictionary and its mention in another document produced in the captaincy, analyzed below, reveals that it was a disease generalized among slaves in the captaincy, even though its meeting changed over time.

A hint that quijila influenced the “new, Afro-American culture which was born in this encounter of various types of African inheritance with the slaveholding society of the New World” (Lara, 2021, p.125-126) appears in *Compêndio narrativo do peregrino da América*, by Nuno Marques Pereira (1939) and published in 1728. In his book, from a Catholic point of view, Pereira constantly condemns the African rites and ceremonies which slaves, often with the complacency of owners, performed in the captaincy. He accuses the “Gentiles, who come from Angola and Costa da Mina, of having among them that abuse of the Quijilas” (spelt with a “j”), from which it can be inferred that in Minas Gerais at this moment the belief had come to be shared by Bantu groups from West Central Africa and Sudanese from the Northeast coast, capable of establishing approximations between their cultures of origin and initiating exchanges and interactions, despite the differences and particularities existing among them. According to Pereira, both groups “observed” quijila, “some so strictly, as if it were a Commandment in the Law of God, and they will die before not observing it” and this consists of “not eating game or fish, shellfish, and many other things” (Pereira, 1939, v.1, p.133-134). In establishing a relationship between quijila and the ten Commandment, he echoed the meaning in which the term was used in catechisms in Kimbundu. From the perspective of Catholic doctrine, he condemned quijila as a sin and defined it as “an explicit pact, which these Gentiles make with the devil,” demonizing it, as with any trace of the African rites he identified in the senzalas of Minas Gerais captaincy. By this pact, “some corporal convenience is established on the part of the person carrying it out: such as good success in war, fortune in the hunt, in farming etc.” (Pereira, 1939, v.1, p.134).

It can be noted that a new significance is attributed to quijila and its new function reveals that it had come to fit into the “fortune-misfortune complex” typical of Sudanese religions from Costa da Mina, especially those from Dahomey. This framework was a reinvention which resulted, “in the first instance,” from “the need to face misfortune or the ‘times of difficult experience,’ of which slavery is undoubtedly one of the most extreme cases” (Parés, 2007, p.109-110). It was not difficult for Sudanese and Bantus to amalgamate their cultures since, as stated above, a process of creolization had already occurred in Africa, something which was not an invention of the Americas. In the same form that in Ndodongo, Matamba, and the Congo, there was a fusion of elements of the Jaga, Ambundu, and Kinbundu cultures, the kings of Dahomey, “after the conquest of the kingdoms of Allada and Ouidah in the 1720s, ... adopted a policy of the appropriation of the cults of the conquered peoples,” as a strategy to consolidate their dominion (Parés, 2007, p.107), important conquests to consolidate their role in the slave trade. The novelty, as the changes in the meaning of quijila reveal, was that the merger came to incorporate the beliefs of those from Costa da Mina, who, living apart on the African continent, were put into contact in slavery in the Americas, constructing bridges between their cultures of origin. It was in this new contest, exiled from their lands, that the different groups coming from the center and north of Africa, that they “became truly ‘African’” (Sweet, 2007, p.142), a notion that did not exist on their continent of origin.

Pereira (1939, v.1, p.134) clarifies that “this Quijila, or pact, passes by tradition to children, grandchildren, and further descendants,” from which it can be observed that in the context of the Americas, Ambundu transmission by lineage was revived, as it was more difficult to count on the presence of ngangas. In eighteenth century Minas Gerais, under the impact of and molded by the experience of slavery, quijila added elements of the original Jaga rite – one of the ijila prescribed food taboos – from the Ambundu culture structured on lineage; and from Dahomean religions, based on the fortune-misfortune complex.^2^ However, it was not only this, in this game of constant reinvention in the captaincy, quijila ended up being transformed into an illness, with clear symptoms and a restricted profile of those who suffered from it.

## Quijila as an illness

According to Antonio Cialli (16 jun. 1749, n.29, f.6v), Quijila “was a really horrible and incurable disease, very frequent among Blacks ..., with true cancerous wounds [*sic*], called Fagederas,” that is wounds with dead flesh or corrosive ulcers. He justifies not going into greater depth because she says that he “will discuss it in a treatise I have in my hands about the endemic and specific diseases of the Americas” (Cialli [Romano], c.out. 1749, f.3-3v). Unfortunately, this text has been lost.

The first case that he described, in 1749, affected “a Black, by the name of Antonio, a slave of Manoel Neto Covas,” who was “full of sores all over the body.” On arriving at Filipe Rodrigues’ plantation, the closest to Lagoa Grande, situated in Minas Gerais captaincy, in 1742, after “washing himself [with its waters] various times during a two-month period, he found himself healthy” ([Cialli], 1749, p.8). He was found by a freed mulatto who “one day was going hunting to entertain himself, and reaching the road to rio das Velhas, and noticed groaning among the undergrowth, a little removed from the road, a Black man.” This was Antonio, who had been expelled by his master, from the “plantation and slavery, as he was incapable of working due to illness ... commonly called = quigila” [written with a “g”] (Cialli [Roman], out. 1749, f.3). The conditions slaves faced were terrible: hard labor left then sick and incapacitated and if they were unable to work, their masters got rid of them, making their survival almost impossible.

Next, “moved by Christian charity the freed mulatto brought the Black man on his horse to the nearest plantation, more to remind him of the name of Jesus than to cure him.” This was the land of Filipe Rodrigues, where they placed him “in a house in which the corn was usually kept” but, “as the fetidness of the sores were unbearable, they told him to wash himself meticulously in the Lake, whose waters they found warm, and they did not hesitate to put the sick man in such an ample space” (Cialli [Romano], c.out. 1749, f.3). To the surprise of all, “within a few weeks of these baths, they noticed that he was stronger and the sores had another appearance, until they finally admired the improvements, and within a few months, he was completely free and this horrible disease was extinct” (Cialli [Romano], c.out. 1749, f.3).

After Father Antonio de Miranda visited the plantation, the news about the curative virtues of the waters of lagoa Grande began to spread. At the end of February 1749, the cleric reported in Sabará, amongst other things, the curing of the slave Antonio’s quijila and the news spread rapidly among the nearby slave plantations. Reaching the lake on 19 March, Cialli (16 jun. 1749, n.25, f.6) found there some people suffering from the illness bathing there and revealed that “their fingers were already consumed, clearly healing,” revealing that those who came to the lake to cure themselves of the disease were more numerous, though his manuscripts mentioned only the first of those who were cured there. It should be noted that there was a significant number of slaves from the Real Extração dos Diamantes (basically the Royal Diamond Mine), sent from Tejuco by the doctor of the local hospital, to bathe in its waters, but it is not possible to know the diseases which affected them (Furtado, 2022a, p.135).

In May, as well as Antonio, another five people suffering from quijila showed marked improvements. These were, (1) Antonio, the slave of Lieutenant Manoel Teixeira Lomba, whose hands and feet were crippled by the disease, but who, after bathing in the lake, was capable of walking; (2) the Black freedman Francisco Xavier Barreto, a resident in Funil, near rio das Velhas, whose “hands were crippled” and which, “after washing a few times, were almost naturally undone;” (3) Jorge, a slave of Jacinto de Sá, who found himself incapable of working since the illness had attacked his feet and eaten his toes, the reason for which his master had expelled him from the house, but who “after bathing himself for a month, found his sores healed, and almost all closed;” (4) Paulo, the slave of Antônio Carlos Moreira, a resident in vila real de Sabará, who had suffered from the disease for seven years, and who had “the beginning of quigila on his left leg,” with the lower part, beside the tibia, being very swollen. The illness, “like termites,” had attacked one foot which, covered with wounds, had almost separated from the leg, but 17 days of bathing were sufficient for it to deflate and to return “almost to natural” size; (5) Manoel, a slave of Manoel Rodrigues, who had been in Brazil for 17 years, suffering from the disease for 30 years, as it had begun in Africa. First, his metatarsals were torn, after some of his toes fell, with the rest being very swollen. After bathing for “a month and a half more than half” of the sores were closed ([Cialli], 1749, p.12, 13, 20-21).

Based on these descriptions, it can be seen that in Minas Gerais captaincy quijila was transformed into a disease which attacked only Blacks, regardless of their origin, whether in Africa or Brazil. Although Black women were not listed among the ill, they were not immune to the disease. Its symptoms were swelling of the limbs – arms and legs –, the appearance of wounds which did not heal on the extremities – on the hands and feet – and, at a more advanced stage, the loss of fingers and toes. Cialli did not describe the origin of the illness, but the Brazilian Antonio de Morais e Silva’s Portuguese dictionary, published in 1789, helps to fill in some gaps and shows that quijila was still in transformation in Brazil. His dictionary not only updated Bluteau’s, which served as its basis, but also incorporated several terms that emerged or were current in Brazil, explaining their meanings.

In the relevant entry quigila (with a “g”, as Bluteau and Cialli had spelled it) is defined as the “antipathy which the Blacks of Africa have to certain food or actions, so that, if they go against this, they suffer disease and perhaps even death follows.” Once again there is no reference to the disease being limited to natives of Angola, nor does it distinguish the place where this spread, as it could be Africa, the New World, and even Portugal, to where the African slaves were sent. Nor are the types of prohibited food specified, while he adds that “some say that these antipathies come from their parents and those who contravene them, come from another world to that of their souls” (Morais e Silva, 1789, p.277). Reverberating its original meaning, the transformations that occurred in Africa, and adding what Cialli observed in lagoa Grande among those affected by the illness, quijila had become both a food prohibition and a disease resulting from the breaking of the taboo. The role of parents remained determinant, dispensing the intermediation of African priests, more difficult to be found or to carry out their functions in the context of colonial slavery. Now, parents appeared in the form of souls from the other world, also reflecting the almost impossibility of Africans to maintain familiar unity after crossing the Atlantic as slaves. This role of the dead was central in the cosmology of groups from Central West African, who saw the universe as divided between the world of the living and of the dead, but with fluidity existing among them. Dead relatives continued to watch over and interfere in lives of their living relatives, even being responsible for imposing social and moral obligations and “at times the spirits of ancestors invoked diseases as a form of castigating those who failed with their family obligations” (Sweet, 2007, p.128-129). Slavery and the Atlantic slave trade shaped the changes operated in quijila at the end of the eighteenth century. Quijila was transformed into a “punishment-disease,” since it came to be “the necessary consequence of what the individual or group provoked,” a “sanction which directly resulted in the transgression of a law,” in this case the alimentary taboos prescribed since Africa (Laplantine, 1991, p.228-229).

The novelty that Cialli and Morais e Silva show is that in Brazil quijila was transformed into a disease and, even though it only attacked Blacks, belief in its existence was shared among masters and slaves, the former exemplified in the doctor and dictionarist and the latter in the Blacks who looked for a cure in the lake. In the dictionary entry, under the rational enlightenment spirit, quijila was framed in the system of sympathies and antipathies, characteristic of Hippocratic-Galenic medicine, inserting this, in the same form as Cialli had done, in the epistemological foundations on which European knowledge was structured. Western culture appropriated, but at the same time modified, the Afro-American one forming in the New World, to adjust it to the European manner of comprehending the world, which can be understood as a process which I call “inter-translation.” This concept starts from the principle that transformations are operated while ideas (knowledge) circulate, either from where they start or where they arrive, when they are inter-translated and modified and thus molded to local conditions. When they adapt to new cultures, the same ideas irradiate again, even reaching the places from where they started, modifying in turn the culture which had forged them. This is what can be observed in the concept of quijila, which under the impact of Portuguese expansionism and colonial slavery, was transformed both in Africa and Brazil, undergoing transformation on both sides of the Atlantic, amalgamating itself as an original Afro-American culture which, unsurprisingly, also incorporated European elements and was shared by African slaves.

## The cure of quijila: inter-translated knowledge

Belief in the power of the waters of lagoa Grande to cure quijila, widely shared by the Blacks who headed there, their masters who allowed them travel, and the doctors who recommended the bathing, is also exemplary of the process of “inter-translation” which characterized this new original Afro-American culture.

In the Bantu cultural universe, “water was the element which divided the world of the living, of the Blacks, from the world of the dead, the whites” (Souza, 2002, p.148). According to Cavazzi (cited by Leguzzano, 1965, v.2, p.214), “in the past, the kings of Angola adored ‘calunga,’ which meant ‘sea’ or ‘great lord’”. They believed in the curative power of water and one of the Ambundu priests, “*Ntinu-a-maza*, which meant ‘king of water,’ ... hid his amulets under the current of a river” (p.96), incorporating its beneficent virtues. The Jaga also adored water and Cavazzi felt that this adoration was “manifested in the veneration they demonstrated for rivers and lakes” (p.215). He states that “as soon as they discover water from afar, they prostrate themselves on the ground to worship it, address prayers, oblations, and vows to it in order not to be bothered by it, or to suffer penalties, not to succumb to diseases, and to be helped with their difficulties” (p.215). Most important among their sacred places was the great lake of Saxia, located on the Upper Cuanza river, in the extreme west of Angola, beside the kingdom of Malemba, where Cavazzi saw with his own eyes these rites of adoration practiced among the local populations (p.215).

In European Hippocratic-Galenic medicine, water was also considered an element with a strong healing power, a fundamental part of the human body, and seen as healthy for all organs. This tradition went back to Western medicine practiced since Antiquity, with the Roman baths being famous. The idea of sympathy and antipathy among the four elements which composed nature and living bodies was a fundamental aspect of this theory and belief in the healing power of water followed the sympathetic path, due to the effects of the vapor from boiling water on the human body, which restored health and combated diseases. In Portugal and in Minas Gerais captaincy various sources of medicinal water were discovered in the eighteenth century (Furtado, 2019, p.76-83), since the European medical theories then in force, including curismo (popular medicine), thermalism, and hydrotherapy, preached the curative value of water. Chernoviz’s pharmacopeia, which circulated widely in Brazil in nineteenth century, identified the mineral waters of Brazil, Portugal, and Europe in a long entry, classifying them in types according to the curative elements present in them. Those of lagoa Grande, already called lagoa Santa, were classified as “simply thermal waters,” with it being pointed out that “they are always warm” (Chernoviz, 1996, v.1, p.274-290), as Cialli pointed in his texts.

It can be observed that the belief in the reparatory power of water was shared by European and African cultures, which explains why masters and slaves, coming from Minas Gerais and other captaincies (sick people came from Bahia, São Paulo, Goiás, and even Pará) sought healing in the waters of lagoa Grande and, in a process of inter-translation, redefined in joint manner the comprehension and healing of quijila. For Europeans, more than any others, the lake symbolized the lavishness with which Divine Providence had blessed its waters. For Africans, it was one of the lakes where their gods “took shelter in its waters” (cited by Leguzzano, 1965, v.2, p.215). If the souls of their ancestors and their curses were capable of crossing the Calunga Grande, as they called the Atlantic, and causing illnesses, so too could their gods and beliefs which protected their bodies already battered by the harshness of slavery.

## Final considerations

In *Gazeta Médica da Bahia*, on September 25, 1866, the doctor Julio Rodrigues de Moura, who worked in Rio de Janeiro, in a letter to the editor, considered quijila (with a “j”), which for him was only its “common name,” a disease of progressive muscular atrophy, associated with elephantiasis due to its “characteristic symptoms,” amongst which was “cutaneous anesthesia” (Moura, 25 set. 1866, p.68-70). For this he drew on the statement by Doctor Antônio José Alves, professor of the Faculty of Medicine of Bahia, best known as the father of the poet Castro Alves (Costa e Silva, 2006, p.9-15), who was doing a study of the disease.^3^ Alves pointed to the disease which began with the appearance of whitish spots, “in whose area the tissues slowly atrophy,” leaving the “skin insensitive.” At a “slow pace, it ends up compromising the respiratory apparatus and the gastro-intestinal tube.” The “complications” associated with it, especially the “ulcerations of the larynx, diarrhea, and pneumonia,” finally result in the “misfortunates affected by it succumbing.” In turn, Alves identified quijila as being elephantiasis, showing that it is “very frequent in Bahia, where it primarily attacks the Black race. And where it is known by [its] African name” (Moura, 25 set. 1866, p.69). At least the African root of the word is remembered! Although Alves recognized that it was more frequent in those he identified as being from the “Black race,” he did not grasp the fact that the reason for this resided in the culture of origin of those affected, where quijila found its meaning. He was more concerned in fitting the disease into the nosological framework of western erudite medicine.

Although it is outside the chronological focus of this article, it is still instigating to show that in the seventh edition of the Morais e Silva dictionary, from 1878, “improved and increased with the large number of new words used in Brazil,” as well as its meaning given in the 1789 edition, states that the noun “quigila” (with a “g”) was already used with the generic, “familiar,” meaning of “antipathy, aversion,” while it is also added that it was a “word from Ambundu language” (Morais e Silva, 1878, p.540). However, in other dictionaries from that time, the Africanness of the term was not even noted. The framing of quijila in Western erudite medicine gradually changed its meaning, eradicating the Central African roots of the disease, because since Hippocrates, symptoms and not the place or culture of origin of sick people is what forms the basis of the nosological framework. With the passing of time the term stopped even referring to a disease, instead resonating the original sense of taboo or prohibition. Luís Maria da Silva Pinto’s dictionary, published in 1832 in Ouro Preto, came to define “quigilha” (with “g” and “lh”), generically, as “the same as antipathy” (Silva Pinto, 1832, p.quig). While in the work of Caldas Aulete, published in Lisbon in 1881, “quisillia” (with an “s” and “ll”) is registered as “antipathy, enmity, anger ... quarrel, something unresolved,” feelings aimed at someone; and as “annoyance, impatience, malaise,” which refer to a thing or a situation: “this makes me quisilia.” There also emerged the verb “quisilar”, meaning to “cause *quisilia* to, to anger, to bother, to upset ... to enrage” and the adjective “quisilento,” meaning “prone to quisilia, which causes quisilia” (Caldas Aulete, 1881, p.1458). This new meanings were a pale reminder of its original meaning among the African jagas.

Between the seventeenth and nineteenth centuries, quijila was transformed between Africa and Brazil, as a result of the African internal dynamics, the advance of Europeans on the continent and, finally, the conditions imposed by the slave trade and the hardships of slavery in Americas. In an inter-translated dialogue of memories and forgetfulness, on both sides of the Atlantic, Europeans, Brazilians and Africans shared and shaped a new culture and quijila was an example of the limits, possibilities and impossibilities of their cultural dialogues.

## NOTES

1 As soon as they sailed along the entire African coastline, in the fifteenth century, the Portuguese identified three large socio-cultural complexes which they used to differentiate the populations they came into contact with along the coastline of Black Africa, a division which is transported to the maps of the continent. The first coincides with Costa da Mina; the second with Congo-Angola-Benguela complex and reaches the East Coast in the region called Costa da Cafraria; and the third corresponds to the Northeast coast, occupied by Swahili population, amongst whom Arab influence was strong (Furtado, 2021, p.131-147). More recently, ethnolinguistic studies have identified the first two groups as Sudanese and Bantus, and although this classification can be criticized, as it is exogenous and produced from the European point of view, it continues to be operational in the historiography.

2 It is worth mentioning here, although its analysis is outside the scope of this article, that in Africa-Brazilian cults which mix religious elements from different parts of Africa, even today there prevail alimentary prohibitions named as quizilas. In this case, see Quizilas… (7 mar. 2017).

3 It should be noted that José Alves’ second wife had been married initially to a slave trader in Bahia and during her marriage to Antônio José Alves, she continued to operate the business of her first husband, even after the international prohibition of the slave trade. “It is not surprising that the profits from the slave trade contributed to the well-being of the Alves family” (Costa e Silva, 2006, p.22).
